# Vertex coloring of graphs via phase dynamics of coupled oscillatory networks

**DOI:** 10.1038/s41598-017-00825-1

**Published:** 2017-04-19

**Authors:** Abhinav Parihar, Nikhil Shukla, Matthew Jerry, Suman Datta, Arijit Raychowdhury

**Affiliations:** 1 Georgia Institute of Technology, Atlanta, GA USA; 2 University of Notre Dame, Notre Dame, IN USA

## Abstract

While Boolean logic has been the backbone of digital information processing, there exist classes of computationally hard problems wherein this paradigm is fundamentally inefficient. Vertex coloring of graphs, belonging to the class of combinatorial optimization, represents one such problem. It is well studied for its applications in data sciences, life sciences, social sciences and technology, and hence, motivates alternate, more efficient non-Boolean pathways towards its solution. Here we demonstrate a coupled relaxation oscillator based dynamical system that exploits *insulator-metal transition* in Vanadium Dioxide (VO_2_) to efficiently solve vertex coloring of graphs. Pairwise coupled VO_2_ oscillator circuits have been analyzed before for basic computing operations, but using complex networks of VO_2_ oscillators, or any other oscillators, for more complex tasks have been challenging in theory as well as in experiments. The proposed VO_2_ oscillator network harnesses the natural analogue between optimization problems and energy minimization processes in highly parallel, interconnected dynamical systems to approximate optimal coloring of graphs. We further indicate a fundamental connection between spectral properties of linear dynamical systems and spectral algorithms for graph coloring. Our work not only elucidates a physics-based computing approach but also presents tantalizing opportunities for building customized analog co-processors for solving hard problems efficiently.

## Introduction

The semiconductor industry is pivoted upon the Von Neumann computer architecture which implements the “Turing Machine” model of computation with a clear distinction between processing units and memory. Computation is carried out through a sequence of instructions with periodic loads and stores to the memory. On the contrary, computation in nature, our brain included, follows a radically different approach. Processing is distributed in all parts of the machine; memory and processors are integrated; clear distinguishable atomic instructions are replaced by continuous time dynamics; and information is encoded in physically meaningful quantities instead of their symbolic interpretations. In spite of the success of the von Neumann computing architecture, its limitations become apparent^1,2^ when dealing with certain classes of problems such as associative computing^3,4^, optimizations^5^, pattern matching and recognition^6^. This has motivated active research in alternative computing models, where dynamical systems have been shown to provide a fundamentally new platform to address these increasingly important problem classes^7–11^.

In this paper, we report experimental evidence and the corresponding theoretical foundation for harnessing the continuous time dynamics of a system of coupled relaxation oscillators to solve vertex coloring of a random graphs, a combinatorial optimization problem of large-scale importance. Combinatorial optimizations represent a problem class where an optimal value of a function, or its optimal point, needs to computed within a domain set which is discrete or combinatorial. Vertex coloring of graphs is a combinatorial optimization problem which is NP-hard (non-deterministic polynomial-time hard), unless P = NP. This means that the best algorithms end up searching the whole domain set for at least some problem instances. Vertex coloring is also one of the most studied NP-hard combinatorial optimization problems not only for its significance in computational theory but also for its many real world applications like fault diagnosis^12^, scheduling^13–15^, resource allocation^16^; Such problems are believed to be solved, or approximated, efficiently in natural processes because they can explore the solution space in a massively parallel manner^17^. In fact, for any deterministic system to be able to solve such hard problems, be it a sequential deterministic Turing machine or a continuous time dynamical system, exponential resources are required which can be in terms of time, hardware components, maximum magnitudes of variables or their precision^7,18–20^.

In the last three decades, dynamical systems as well as hardware implementations have been proposed to solve NP problems, many of which implement some form of algorithm for solving, or approximating, such problems. Important attempts include Quantum computers^21^, Cellular automata^22^, Hopfield networks^5^, Ising model formulations^23^, chaotic nonlinear attractor systems^7^, iterated projections^24^, Memcomputing^25^ and stochastic searching using non-repeating phase relations among oscillators^26^. All these approaches, except iterated maps, are based on the idea of interconnected “nodes” which exchange, store and process information among themselves. One particular variant of such dynamical systems is based on a network of coupled oscillators whose phase and frequency dynamics can be exploited to encode system states^3,27,28^. These computational kernels, which have been claimed to mimic the spiking networks of the human brain, have been successfully used in associative computing, demonstrating significant improvements in energy-efficiency and performance when applied to video analytics^29^. Theoretical models of coupled sinusoidal oscillators, (*Kuramoto* models^30,31^), or van der pol oscillators^32,33^ have often been used to theoretically study asymptotic limits; but their physical implementations and experimental evidence of such networks to solve computationally hard problems have remained elusive.

**Figure 1:**
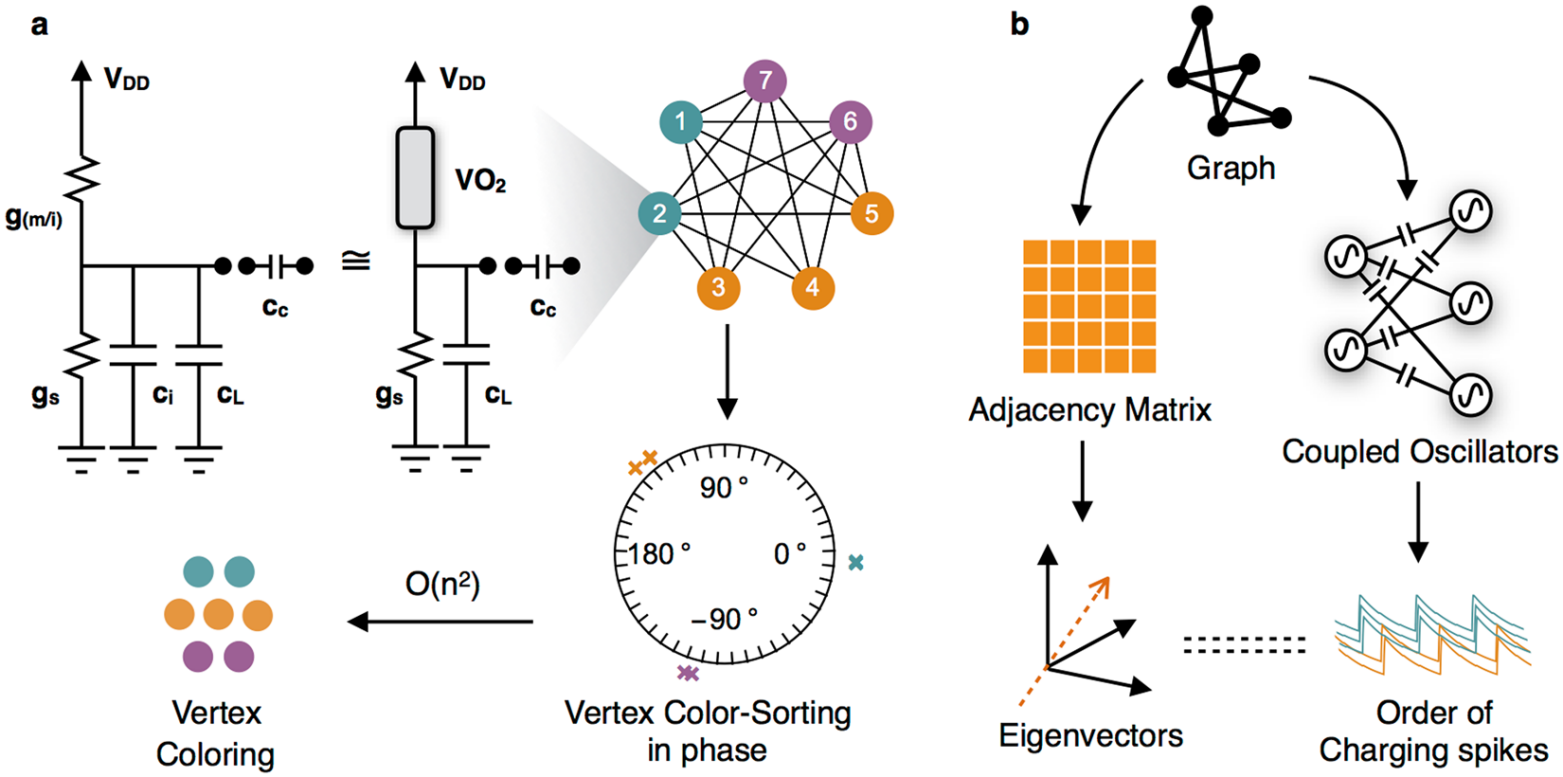
Overview of the circuit and system dynamics. (**a**) Overview of the proposed system for vertex coloring and a simulation example. First step is a coupled relaxation oscillator circuit where the oscillators are composed of a series combination of VO_2_ device and a resistor (with a loading capacitor in parallel), and are connected in a graph using capacitors. The equivalent circuit diagram of the VO_2_ oscillator is shown using an internal capacitance *c*_*i*_ and a phase changing conductance *g*_(*m*/*i*)_ which switches between metallic conductance *g*_*m*_ and insulating conductance *g*_*i*_. An example 3-partite graph is simulated and the relative phases of these oscillators are shown in a phase diagram which shows vertex color-sorting in phase, and can be used to calculate vertex-coloring with O(n^2^) complexity. (**b**) The circuit is composed of VO_2_ oscillators capacitively coupled in a network same as the input graph. The final order of phases, or charging spikes, of the oscillators is related to the eigenvectors of the adjacency matrix of the input graph which in turn are related to the solution of the graph coloring problem.

**Figure 2:**
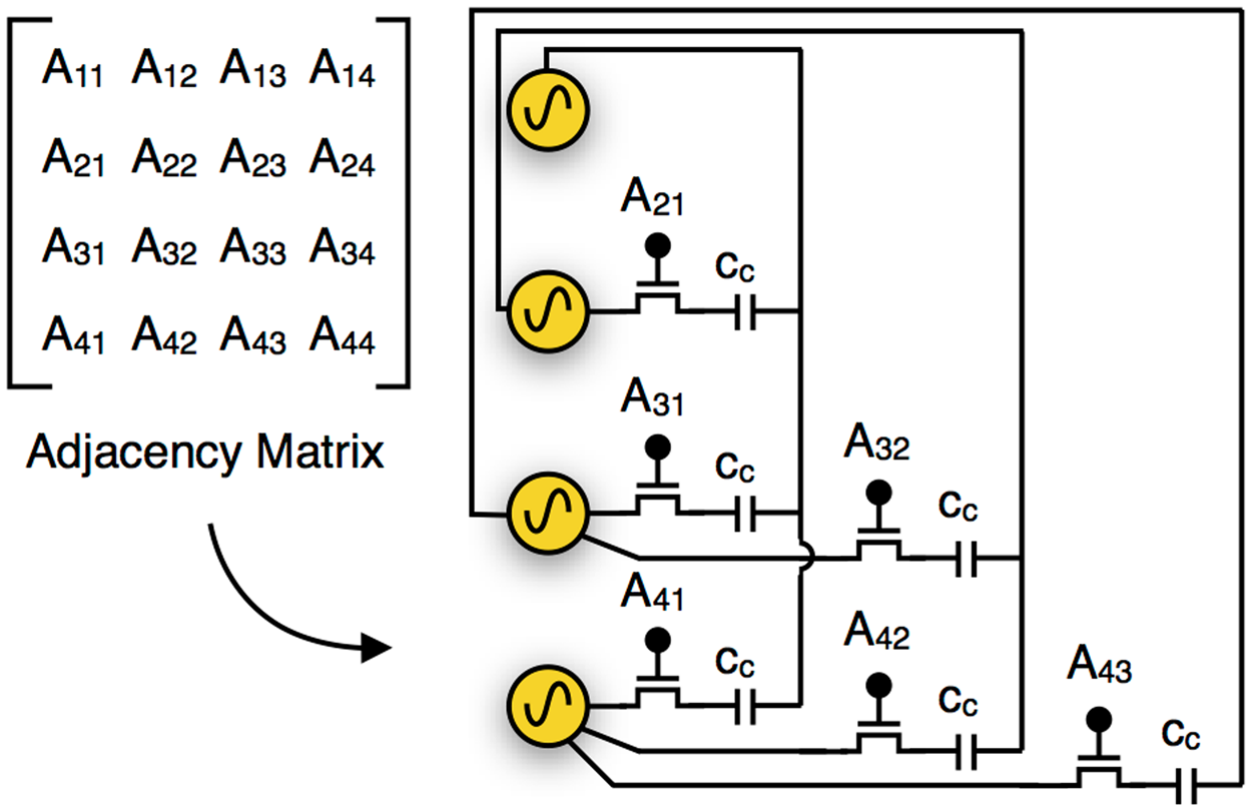
Coupled oscillator circuit schematic. A circuit of 4 coupled oscillators with capacitive connections between oscillators controlled using switches corresponding to the adjacency matrix *A* and coupling capacitance *c*_*c*_. The subscripts denote the corresponding entries in *A*. Note that *A*_*ij*_ = *A*_*ji*_, *A*_*ii*_ = 0 and *A*_ij_ ∈ {0,1}.

Here, we establish that a system of coupled relaxation oscillators fabricated using Vanadium dioxide (VO_2_) metal-insulator-transition devices and coupled capacitively, can lead to system dynamics on which vertex coloring of unweighted and undirected graphs (hitherto referred to as the graph coloring problem) can be successfully mapped (Fig. 1a). A VO_2_ oscillator consists of a VO_2_ device connected in series with a conductance *g*_*s*_ (with a loading capacitor *c*_*L*_ in parallel) and the output node is the node between the VO_2_ device and the conductance (Fig. 1a). Such a simple series circuit shows self-sustained relaxation oscillations. In previous works by authors, properties of two such coupled Vanadium Dioxide (VO_2_) based relaxation oscillators have been analyzed^28,34^ and pairwise coupled circuits have been proposed for basic computation tasks^3,35^, but using complex network connections with VO_2_ oscillators (or any other kind of oscillators) for more complicated computing tasks have been challenging in theory as well as in experiments. We demonstrate experimentally and using simulations that when such relaxation oscillators are coupled using only capacitances in a manner topologically equivalent to an input graph, their steady state phases can be used to approximate the solution of the NP-hard *minimum graph coloring problem*. For this, we propose a reformulation of the graph coloring problem where instead of finding a color assignment for each node, the objective is to find a circular ordering or circular permutation of the nodes such that the same colored nodes appear together in the ordering. Such a reformulation preserves the hardness of the problem and is useful for interpreting the output of our circuit (Fig. 1a). We show analytically that the dynamics of such a coupled relaxation oscillator system is intrinsically connected to spectral algorithms for graph coloring^36–38^, which use eigenvectors of adjacency matrix of the input graph to approximate the solutions. Alternatively, the permutation of steady state phases of coupled relaxation oscillators depends on eigenvectors of the adjacency matrix in the same way as have been used by spectral algorithms for graph coloring (Fig. 1b). A programmable circuit for a such a coupled oscillator system, where the oscillators are coupled in a graph with adjacency matrix, *A* and coupling capacitance *c*_*c*_ is shown in Fig. 2. Such an all-to-all connected circuit can be laid out in 2-dimensions with each connection controlled by a switch (transistor). The gate voltages of transistors can be used to provide the input, viz. the adjacency matrix *A* of the problem graph instance. Our simulation results show that the hardness of problem instances has, on average, expected effects on important metrics of solutions found using such a circuit like the number of colors detected and the settling time.

It is well known that eigen properties of the coefficient matrix in the evaluation equation of a dynamical system determine important structural properties of the system including stability, bifurcation, energy minima(s) and overall system dynamics. In this report, we provide a theoretical bridge and experimental evidence that a dynamical system whose coefficient matrix inherits properties of the incidence matrix of a graph, can indeed emulate spectral graph algorithms just through its time evolution. We envision such dynamical systems to provide foundational paradigms in the development of next-generation computational accelerators and kernels.

## Results

### Minimum Graph Coloring Problem and its reformulation

The objective of *graph coloring* or *vertex coloring* is to assign one color (out of total *k* colors) to each vertex of an undirected graph such that no two adjacent vertices receive the same color. A graph coloring that minimizes the number of colors *k* is called *minimum graph coloring*. The minimum *k* for which a correct coloring is possible is called the *chromatic number* of the graph. A graph which can be colored using at most *k* colors is called a *k-partite graph*. A *k*-partition of a set, like the set of nodes, is a grouping of the elements of the set into *k* groups. Hence, a vertex coloring with *k* colors is a *k*-partition. We reformulate the objective of finding a color assignment to finding a circular permutation of nodes such that the same colored nodes appear together. We refer to this reformulation as *vertex color-sorting* and the corresponding optimal version as *minimum vertex color-sorting*. Calculating a color assignment from a *color-sorting* is *O*(*n*^2^). This is because if *P* is the permutation matrix for the color-sorting and *A* is the symmetric adjacency matrix of the graph, then the color assignments can be found by observing the ‘0’ diagonal blocks in the matrix *PAP*^*T*^. This makes *vertex color-sorting* as hard as *vertex coloring* (see Supplementary Section 4). Using this method, any permutation *P* gives a correct color assignment but a *better* permutation gives lesser number of colors, and an optimal color-sorting permutation gives the minimum number of colors. In the proposed coupled oscillator system, each oscillator represents a vertex (or node) of the graph. Any two nodes connected in the original graph by an edge (as indicated by a ‘1’ in *A*), are capacitively coupled in the hardware implementation. As the coupled system evolves, the relative phases of the oscillators are ordered, and we observe that the relative ordering of the phases approximates *minimum vertex-color-sorting* of the original graph.

### Experimental Demonstration of Vertex Coloring in a Coupled Oscillator Network

We construct a relaxation oscillator by exploiting the electrically induced large and abrupt change in resistance across the insulator-to-metal transition (IMT) in Vanadium Dioxide (VO_2_)^39,40^, and stabilizing it with a negative feedback from a series conductance *g*_*s*_ (details of the single oscillator dynamics have been elucidated in our previous work^28,34^). Since the IMT in VO_2_ is a materials-level manifestation of hysteretic, resistive threshold switching behavior^41,42^ critical to realizing relaxation oscillatory action^43^, VO_2_ based oscillators present a compact, scalable, and potentially low power solution^35^ to realizing the fundamental building block of the graph coloring hardware. Further, we use a non-dissipative capacitive coupling scheme to connect the oscillators and achieve frequency synchronization.

**Figure 3:**
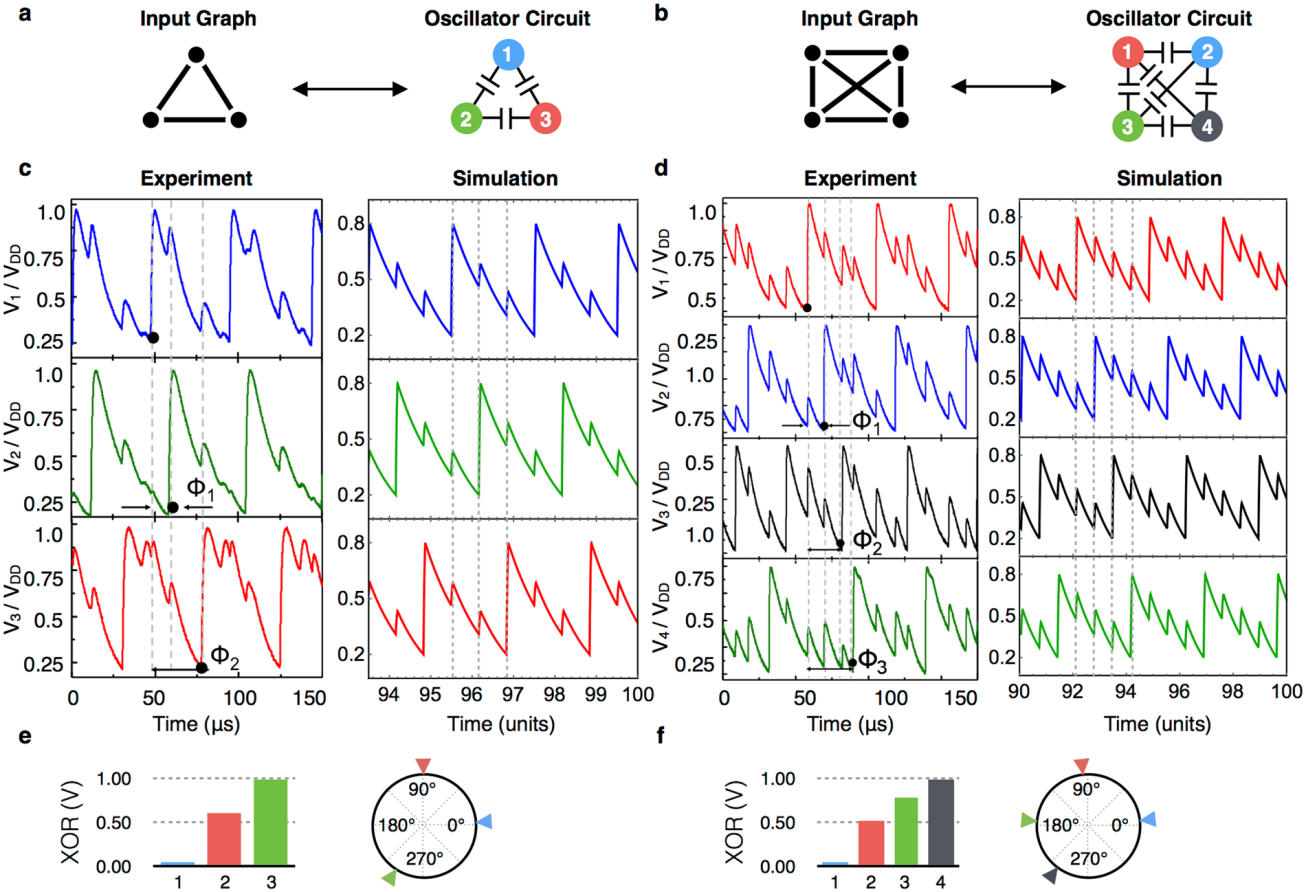
Phase dynamics of synchronized VO_2_ based capacitively coupled relaxation oscillators. (**a,b**) Schematics of two representative configurations (a: delta configuration; b: cross-connected ring) of capacitively coupled VO_2_ based oscillators, and their corresponding graphs. (**c,d**) Time domain waveforms from experiment and simulations for the two coupled oscillator configurations in (**a,b**), respectively, showing that while the oscillators are synchronized in frequency, no two directly coupled oscillators are in-phase. This important property of the coupled oscillator system enables graph coloring. (**e,f**) Time averaged XOR of thresholded outputs of oscillators (each w.r.t. oscillator number 1), and respective polar phase plots showing steady state relative phases detected using PFDs. The XOR values are normalized with respect to the maximum value.

Figure 3 shows two representative configurations of graphs (Fig. 3a: delta configuration; Fig. 3b: cross-connected ring configuration) along with their equivalent implementations using coupled oscillators. The respective time domain waveforms of the oscillators (Fig. 3c,d) reveal a unique relationship among the phases of the oscillators: there is a distinct non-zero phase difference between any two directly coupled oscillators. This is because the nature of capacitive coupling among the relaxation oscillators ensures that two adjacently connected oscillators will tend to force each other out of phase; a rigorous mathematical treatment of the phase dynamics between two coupled VO_2_ based oscillators has been detailed in an earlier work^34^. Additionally, when an oscillator is connected to multiple other nodes, the net phase of the oscillator is the aggregate of the ‘repelling effect’ of all the other connected oscillators. As such, in the light of vertex coloring, such a circuit is expected to result in output phases which are clustered by color, i.e. oscillators with the same color have phases which are close together. But such an interpretation of oscillator phases is weak and is difficult to apply in most cases of graphs where the corresponding circuit output either does not have well clustered phases or have incorrect clusters. Our interpretation of outputs as *color-sorting* solves all these problems and is well defined. As will be discussed in the next section, the combined repelling effect in a network of oscillators gives special properties to the order of phases of oscillators in steady state, viz. they approximate *minimum vertex-color-sorting*. As discussed earlier, this steady state ordering of phases is then used to calculate a *vertex-coloring*.

Since the oscillators in this work are non-sinusoidal in nature, the steady state phase differences among the coupled oscillators can be calculated using phase-frequency detectors (PFDs) or the time-averaged XOR metric^35^. The time-averaged XOR measure of any two oscillator outputs is calculated by first thresholding the outputs to binary valued waveforms and then taking the time average of absolute difference of these thresholded waveforms over the complete steady-state periodic orbit of the system. The time-averaged XOR metric is proportional to the absolute value of phase difference between the oscillators, and hence it does not differentiate between lead or lag. Figure 3e,f show the relative phases detected using a PFD (shown using the polar phase plots) and the XOR measures of each oscillator with respect to a common reference oscillator (shown as bar graphs).

**Figure 4:**
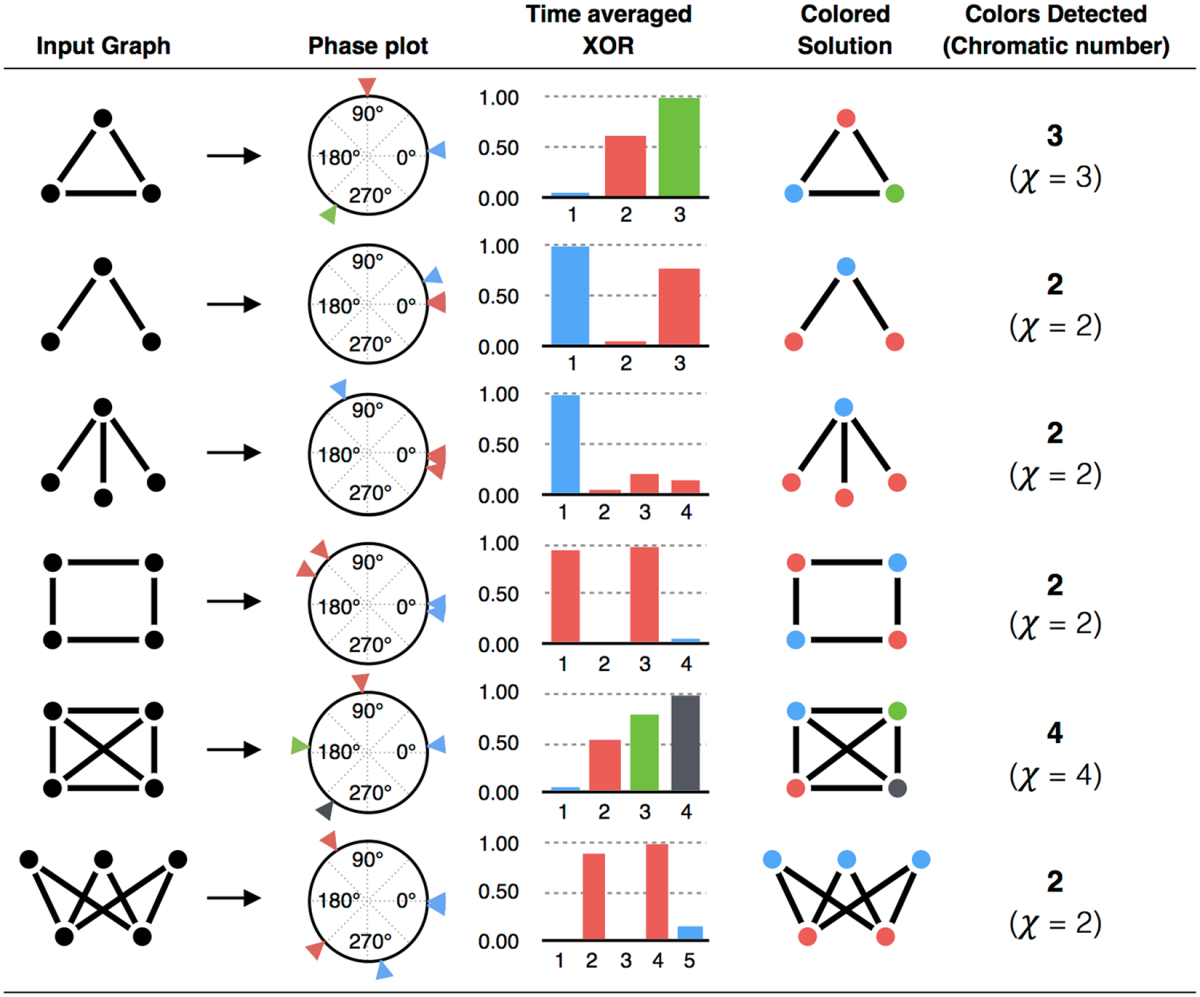
Experimental results of graph coloring using coupled VO_2_ based oscillators. Various graph configurations, and their experimentally obtained solutions (PFD outputs and XOR values) using the coupled relaxation oscillator system. After mapping the graphs onto the coupled oscillator hardware, the steady state order of phases of oscillators is used as a color-sorting and a color assignment is calculated.

Next, we experimentally investigate the coloring of some other graph configurations with up to five vertices, using the system of VO_2_ based oscillators (Fig. 4). The coupled oscillators are configured to represent the respective graphs as discussed earlier, and the corresponding values of the time-averaged XOR along with the respective phase plots of the oscillators are shown in Fig. 4. It can be observed that the hardware is able to *optimally* color all the graphs investigated here.

### Analytical Model, Piecewise Linear System Dynamics and connection to Spectral Algorithms

The mathematical model of the circuit is created taking into consideration the fact that the fundamental switching time constant for the phase transition (i.e., IMT and MIT) in correlated materials like Vanadium Dioxide (VO_2_) occurs on extremely fast time-scales (~75 fs)^44,45^. Even for electrically induced phase transitions, the expected time constants in scaled VO_2_ devices^46^ are a few orders of magnitude smaller than the RC time constants observed in the oscillator circuit. As such, the phase transition is modeled as an abrupt instantaneous switching. The VO_2_ devices switch between a low resistance metallic state with conductance *g*_*m*_ and a high resistance insulating state with conductance *g*_*i*_ based on the voltage *v*_*d*_ across their two terminals. On increasing *v*_*d*_ the device switches to a metallic state (insulator-to-metal (IMT) transition) after a threshold *v*_*h*_, and on decreasing *v*_*d*_ below *v*_*l*_ the device switches back to an insulating state (metal-to-insulator (MIT) transition). Here *v*_*h*_ > *v*_*l*_ and *v*_*h*_ − *v*_*l*_ defines the hysteresis in switching. Consider a supply voltage which is applied across the series combination of such a hysteretic device and a conductance *g*_*s*_ where the subscript *s* denote a series conductance. Without loss of generality, we assume that at *t* = 0 the device is in high resistance state and the voltage drop across the device *v*_*d*_ = 0. The internal capacitance of the device charges up and *v*_*d*_ increases and eventually crosses the threshold *v*_*h*_. Due to this the device transitions into a metallic state which causes the internal capacitance of the device to discharge and reduces *v*_*d*_ which finally drops below *v*_*l*_. This causes the device to switch back to the insulating state resulting in oscillations with piecewise linear dynamics. In case of the coupled oscillator circuit, a loading capacitance *c*_*L*_ of appropriate magnitude is required as shown in Fig. 1a for correct circuit operation.

The dynamics of a single oscillator (Fig. 1a) can be written as the following piecewise differential equation:

1 $$cv^{\prime} (t)=-g(s)v(t)+p(s)$$

where *c* is the lumped capacitance of device along with the loading capacitance and parasitics, *s* ∈ {0,1} is the state of system - charging (denoted by 1) or discharging (denoted by 0), and *g*(*s*) is the net path conductance in state *s*, with *g*(*s*) = *g*_*s*_ + *g*_*i*_*s*. If the voltage *v* is normalized to *v*_*dd*_ then *p*(*s*) = *s*. The dynamics of a circuit of identical coupled relaxation oscillators can be described using the following matrix differential equation:

2 $${\boldsymbol{x}}{\boldsymbol{^{\prime} }}(t)={({C}_{i}+{C}_{c}+{C}_{l})}^{-1}[-G({\boldsymbol{s}}){\boldsymbol{x}}({\boldsymbol{t}})+{g}_{i}{\boldsymbol{s}}]$$

Here, ***x*** is the vector of all voltages (normalized to *v*_*dd*_), *C*_*i*_ is a diagonal matrix with the diagonal elements equal to the internal capacitances of the corresponding oscillator nodes, *C*_*c*_ is the coupling capacitance matrix with diagonal elements equal to the sum of all the coupling capacitances connected to the corresponding nodes and off-diagonal elements equal to the coupling capacitances of corresponding pair of nodes with negative sign, ***s*** ∈ {0,1}^*n*^ is the vector of states of all oscillators, *G*(***s***) is a state dependent diagonal matrix with $diag(G({\boldsymbol{s}}))={g}_{s}+{g}_{i}{\boldsymbol{s}}$, and *C*_*l*_ is a diagonal matrix corresponding to the extra loading capacitors. These loading capacitors effectively add to the internal capacitance and are chosen such that *diag*(*C*_*c*_ + *C*_*l*_) is constant. When all oscillators have equal internal capacitances *c*_*i*_ and equal coupling capacitances *c*_*c*_, then *C*_*i*_ = *c*_*i*_*I* where *I* is the identity matrix, *C*_*c*_ = *c*_*c*_*L*, *L* being the laplacian matrix of the graph with *L* = *D* − *A* where *D* is a diagonal matrix of degrees of vertices and *A* is the adjacency matrix of the graph. In such a case, a simple choice of *C*_*l*_ is *C*_*l*_ = *c*_*c*_(*nI* − *D*). These oscillators have very high charging rate (due to the high conductance of the metallic state of the VO_2_ devices) and low discharging rates (due to relatively high resistance of the pull-down resistors), and as such, the phases of oscillators and their permutation can be read by observing the relative positions of the charging spikes.

**Figure 5:**
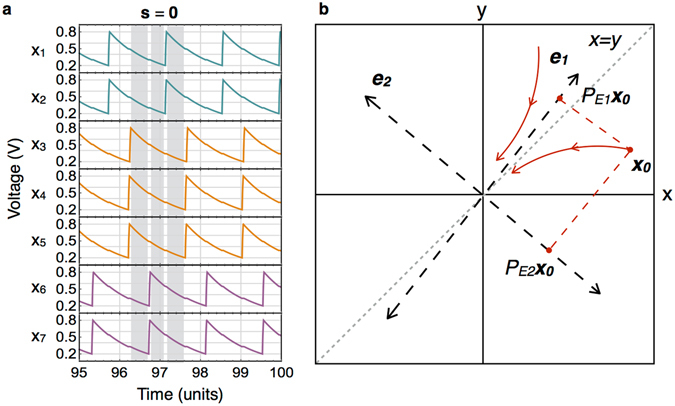
System dynamics and asymptotic permutation. (**a**) Simulation waveforms of a circuit connected in the 3-partite graph shown in Fig. 1a. The gray regions show the time when the system is in state ***s*** = 0. (**b**) Representative figure showing the relation between the asymptotic order of components of the state vector and the eigenspaces in a two-dimensional linear system where the coefficient matrix has negative eigenvalues.

Considering the system of (2), if there exists a limit cycle where the system settles to a certain order of charging spikes, then the order of charging spikes is same as the order of components of the state vector ***x*** in state ***s*** = 0 (Fig. 5a), where the orders are considered unique up to a circular permutation and 0 is a vector of zeros. In other words, orders which are circular permutations of each other are considered same. The system of (2) in state ***s*** = 0 is a linear dynamical system with all negative eigenvalues and the asymptotic order of components of the state vector ***x*** is determined by the asymptotic direction of the system trajectory. A representative figure of system trajectories in such a linear dynamical system in two dimensions is shown in Fig. 5b in which ***e***_1_ and ***e***_2_ are the eigenvectors with distinct negative eigenvalues of the coefficient matrix *B* of a linear dynamical system $\dot{{\boldsymbol{x}}}=B{\boldsymbol{x}}$, where ***x*** = {*x*,*y*}. The eigenspaces *E*_1_ and *E*_2_ are the lines along ***e***_1_ and ***e***_2_ respectively and *P*_*E*1_ and *P*_*E*2_ are projection matrices for these eigenspaces. Considering (2), the eigenvectors of coefficient matrix (*C*_*i*_ + *C*_*c*_ + *C*_*l*_)^−1^ with the least negative eigenvalues are, in fact, same as the eigenvectors of adjacency matrix *A* with most negative eigenvalues (Supplementary Proposition 1). These eigenvectors determine the asymptotic order of components of the state vector ***x*** in the following way. Let *T*(***x***) represent the order of components of vector ***x***. Referring to Fig. 5b, let ***x***_0_ be the initial starting point of the system, *E*_1_ the eigenspace with least negative eigenvalue, *E*_2_ with the next higher eigenvalue and so on. Then the asymptotic order of components is same as the order of components of *P*_*E*1_***x***_0_. In case *P*_*E*1_***x***_0_ has some components which are equal (with respect to Fig. 5b, it means if ***e***_1_ lies along the *x* = *y* line) then the order among those equal components is decided by the order of *P*_*E*2_***x***_0_ and so on (see Supplementary Section 2 for analytical derivations). If this operation of combining two orders is represented by $\oplus ^{\prime}$ where the first order is preferred over the second, then the asymptotic order can be written as:

3 $$Q({{\boldsymbol{x}}}_{0})=T({P}_{E1}{{\boldsymbol{x}}}_{0})\oplus ^{\prime} T({P}_{E2}{{\boldsymbol{x}}}_{0})\ldots $$

The eigenvectors with least negative eigenvalues determine not just the asymptotic order in state ***s*** = 0 but also the limit cycle. To obtain an intuitive understanding of how the system settles to a limit cycle with the correct color-sorting, i.e. steady state oscillations where order of charging spikes become constant and equal to a correct color-sorting, we note that such a limit cycle exists if:

1. The state ***s*** = 0 does not change *T*(***x***) when *T*(***x***) is a correct color-sorting.
2. The charging states ***s*** ≠ 0 change *T*(***x***) by only a circular permutation.
3. The state ***s*** = 0 does not change *T*(***x***) even when *T*(***x***) is a circular permutation of the correct color-sorting for which property 1 holds.

Property 1 is true when the system is at state ***x*** such that *T*(***x***) corresponds to the order determined by the least negative eigenvalues (from (3)) because then it is same as the asymptotic order of components and hence does not change. Also, the eigenvectors with the least negative eigenvalues have a property, as used in spectral algorithms for graph coloring^36–38^, that their components corresponding to same color tend to be equal (or close) for dense graphs, and these components disperse as graphs become sparse. For instance, in a complete 3-partite graph with arbitrary partition, the eigenvectors have exactly equal components for nodes belonging to the same partition subset (color class). Hence, the order determined by these least negative eigenvectors (or eigenspaces) as given by (3) will correspond to a correct color-sorting with minimum number of colors for dense graphs and the number of colors would increase for sparser graphs. This can also be seen in the light of perturbation theory of matrices. Any sparse *k*-partite graph (or equivalently its adjacency matrix) can be obtained from a complete *k*-partite graph with the same partition structure by removing some edges (or changing ‘1’s in its adjacency matrix to ‘0’s). Such a “perturbation” of adjacency matrix has an equivalent effect of rotating its eigenvectors by some angles^47^ which depend on the magnitude of perturbation, which in this case is the number of edges removed. We empirically find properties 1–3 to be true for complete *k*-partite graphs which are the densest *k*-partite graphs (see Supplementary Section 5 for an analytical discussion), and considering sparse graphs as perturbations of complete partite graphs, we can say that vertex color-sorting using the coupled relaxation oscillator circuit becomes less optimal as graphs become sparse. This is known to be true for spectral and other coloring algorithms as well that *k-*colorable dense graphs are easier to color than sparser ones.

### Simulation Results and Performance Assessment

We simulate the dynamical system as described by (2) for random graph instances of 3-colorable graphs. The initial conditions are chosen at random ${{\boldsymbol{x}}}_{0}\in {[0.35,0.65]}^{n}$ and ***s*** = 1 at *t* = 0 because all oscillators are in charging state when the power is switched on. Without loss of generality, *v*_*l*_ and *v*_*h*_ are chosen as 0.2 V and 0.8 V respectively with a supply voltage of 1 V. We use a random graph generation model *G*(*n,k,i*) to generate instances of colorable *k*-partite graphs with total *n* nodes. The graphs are generated by first choosing a random *k*-partition of *n* nodes, then creating a complete *k*-partite graph with this *k*-partition and finally removing random *i* number of edges from this complete graph. *Average connectivity* is defined as the ratio of total number of edges in the generated graph *G*(*n,k,i*) to the total number of edges in the complete *k*-partite graph with the same partition.

As is true with hard problems, even in graph coloring problems no heuristic algorithm works best for all graph instances^48^. Also different heuristics work better for different instances, and hence no single *order parameter* can account for the hardness of an instance of a graph coloring problem^49–51^. The most commonly used order parameter is average connectivity^52^. We use this parameter to account for the hardness of the problems being solved and observe how a coupled relaxation oscillator network behaves for problems with varying levels of average connectivity. Observations are made particularly about the *cluster diameter*, the *number of colors detected* and the *settling time*, which are defined as follows.

When the coupled oscillator circuit settles to a correct color-sorting, the phases of oscillators or nodes with the same color form a cluster for many graphs, esp. the dense graphs. The maximum phase difference of two oscillators in the same cluster, i.e. with the same color, is called the *cluster diameter*. The *number of colors detected* is calculated using the order of charging spikes at the end of the finite time period for which the circuit is simulated. *Settling time* is the defined as the time after which the number of colors detected does not change till the end of the simulation time.

**Figure 6:**
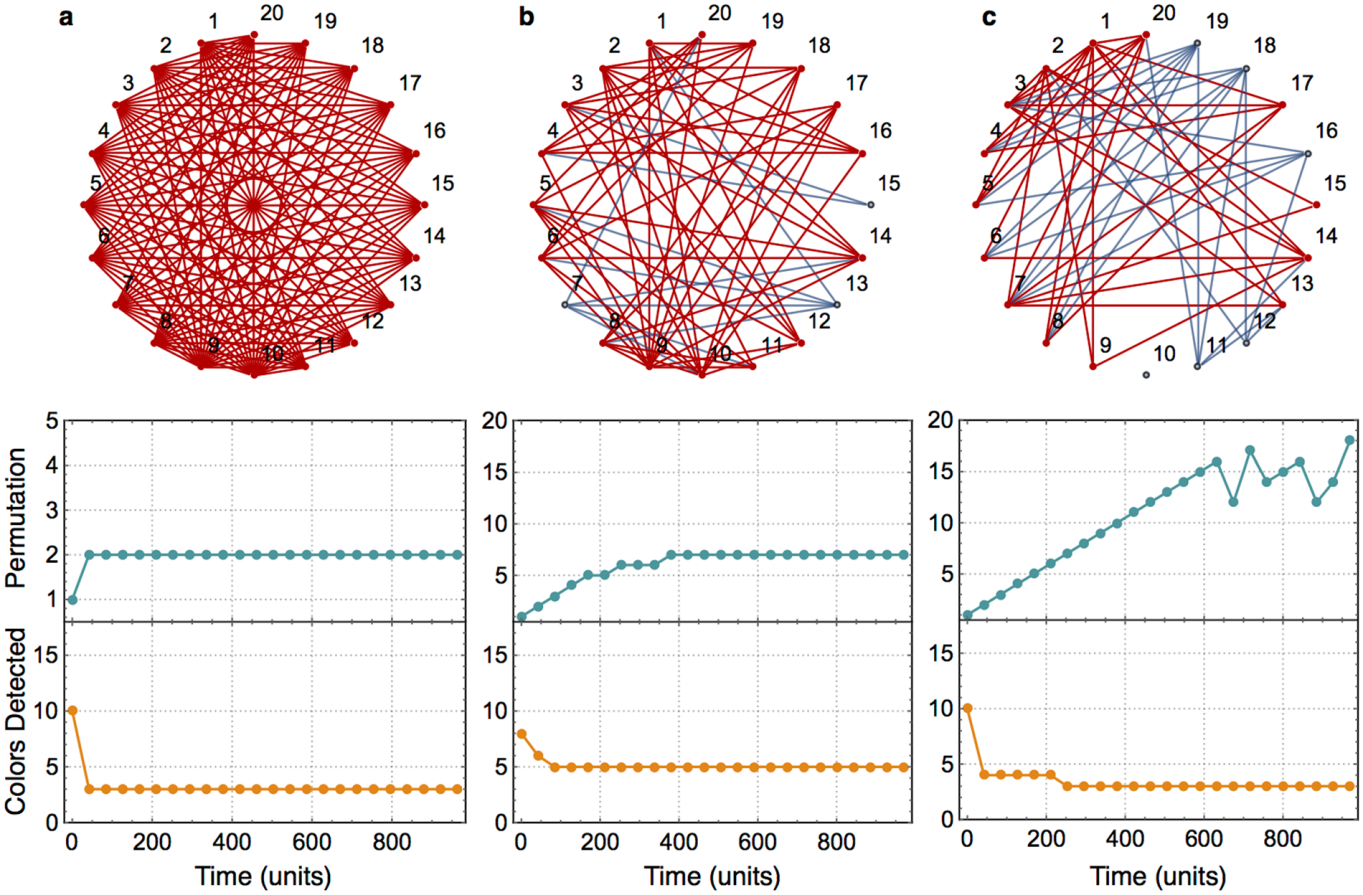
Effect of hardness on circuit behavior. (Above) Graph diagram and (below) waveforms of permutation number (a unique number corresponding to each permutation) of charging spikes and number of colors detected with time. All graphs are 3-partite with partition (8,2,10) with varying levels of average connectivity: (**a**) 1.0, (**b**) 0.57 and (**c**) 0.48. All triangles in the graph are shown with red edges and the rest edges are shown in blue.

Figure 6 gives a visualization to how the order of charging spikes evolves with time for 3 different graphs of 20 nodes with decreasing average connectivity. All three graphs are 3-partite with partition (8,2,10). For a single simulation instance, we note the order of charging spikes at various time instances and associate a unique number (within a simulation instance) to each permutation. A plot of this permutation number with time shows how the order, or permutation, of the charging spikes evolves with time. Figure 6 shows this plot along with plot of the number of colors detected using the order of charging spikes at various times. Figure 6a shows the typical case of a complete partite graph where the order of charging spikes settles quickly to a correct color-sorting, and the number of colors detected falls quickly to the minimum number of colors (3 in this case). Figure 5b and c show graphs with lower connectivity but the same partition structure. We make two observations. Firstly, even after the number of colors detected settles down, the permutation or order of charging spikes can evolve. Secondly, Fig. 6c shows lower number of colors detected than Fig. 6b but the settling time is higher for Fig. 6c. As such, both settling time and number of colors detected can be considered as *imperfect* order parameters for hardness of graph coloring just like average connectivity.

**Figure 7:**
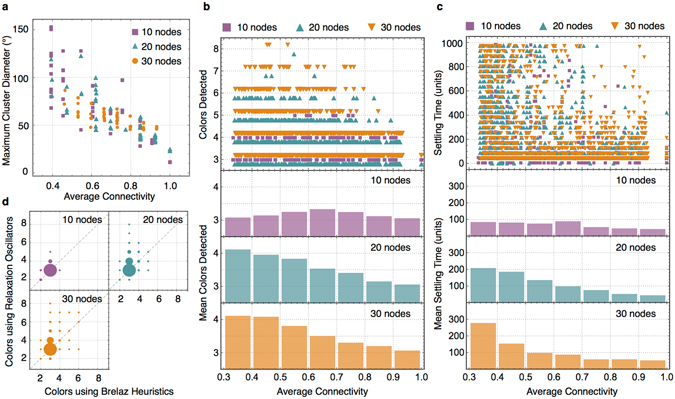
Simulation results on random graph instances. (**a**) Maximum cluster diameter for those graphs for which 3 colors were detected. (**b**) (Top) Number of colors detected plotted against the average connectivity. (Below) Mean colors detected in connectivity intervals. (**c**) (Top) Settling time plotted against the average connectivity. (Below) Mean settling time in connectivity intervals. (**d**) Number of colors detected using the relaxation oscillator circuit plotted against number of colors detected using Brelaz heuristics for the random graph instances used in b and c. Each graph instance represents a point whose coordinates are denoted by the pair of colors detected using the two methods. The size of dots represents the percentage of instances which lie at the center of corresponding dot.

Figure 7 shows the performance of such network on random graph instances. We generate 3-partite graphs using *G*(10,3,*i*), *G*(20,3,*i*) and *G*(30,3,*i*) with increasing values of *i*. The graphs which become bipartite after removing *i* edges are discarded. Each graph instance is used 5 times with a new random initial condition for the relaxation oscillator circuit. Various metrics to evaluate the circuit output are plotted against average connectivity. We see that as graphs become sparse with decreasing average connectivity, the cluster diameter increases (Fig. 7a). This comparison is made only among those graphs where the final phases are clustered correctly into 3 clusters, i.e. the number of colors detected is 3. When graphs are dense and closer to being complete partite, the possibility of them being optimally colored with 3 colors is high, and the settling time on average is less (Fig. 7b,c). As graphs become sparser, the number of colors detected (Fig. 7b) as well as settling time (Fig. 7c) increase statistically on average. It also follows our intuitive understanding that hard computational problems remain hard even under domain transformation, albeit with potential practical implications such as increased energy-efficiency and performance benefits of continuous time systems over their digital counterparts. A comparison of colors detected from simulating the coupled oscillator network with that using Brelaz Heuristics^53^ (Fig. 7d) shows the effectiveness of the circuit as a tool to approximate the *minimum graph coloring problem*. Number of colors detected by simulating sample graphs from the second DIMACS implementation challenge^54^ are shown in Table 1, where for certain instances we note that the dynamical system outperforms heuristic algorithms.

**Table 1:** Comparison with Brelaz heuristics.

Graph	Nodes	Chromatic Number	Minimum number of colors found	
			Brelaz Heuristics	Coupled Oscillator Circuit
huck	74	11	11	12
myciel3	11	4	4	4
myciel4	20	5	5	5
myciel5	47	6	6	6
myciel6	95	7	7	8
david	87	11	11	13
queen5_5	25	5	7	6
queen6_6	36	7	10	12
queen7_7	49	7	12	12
queen8_8	64	9	15	14
DSJC125.1	125	—	8	9
DSJC125.5	125	—	24	34

Comparison of the number of colors detected using Brelaz heuristics with those detected using a coupled relaxation oscillator circuit for various graph instances from the second DIMACS implementation challenge.

In this article, we have established that a system of capacitively coupled relaxation oscillators can perform graph coloring, which is a commonly studied and practically useful combinatorial optimization problem. Further, the connection between system dynamics and the order of steady state phases of oscillators with spectral techniques for graph coloring has been discussed and it shows an innate, yet, hitherto unexplored, connection between the time evolution of dynamical systems and computationally hard problems that have solutions or approximations in the spectral domains.

## Methods

### Experiments with VO_2_ devices

#### Growth

The VO_2_ films have a thickness of 10 nm, and are epitaxially grown on (001) TiO_2_ using reactive oxide molecular beam epitaxy. The epitaxial mismatch between VO_2_ and TiO_2_ results in a tensile biaxial strain of −0.9%.

#### Two-terminal VO_2_ device fabrication

The electrodes are patterned using contact lithography followed by electron beam evaporation of Pd/Au (20 nm/80 nm) and lift-off in RemoverPG at 70 °C. Next, the channel width and isolation are defined by electron beam lithography followed by a CF4 dry etch. Finally, the resist is stripped with RemoverPG at 70 °C.

### Circuit simulations of coupled relaxation oscillators

The oscillator circuits were simulated in Mathematica 10.2 for a finite time (1000 time units). Simulations were performed using default settings for NDSolve routines. The metal-insulator transition events were detected using the inbuilt Mathematica event detection in NDSolve routines with default settings. For Brelaz heuristics, Mathematica routine for Brelaz heurstics was used.

## Electronic supplementary material

Supplementary Text
