# Author Correction: Vertex coloring of graphs via phase dynamics of coupled oscillatory networks

**DOI:** 10.1038/s41598-018-23427-x

**Published:** 2018-04-12

**Authors:** Abhinav Parihar, Nikhil Shukla, Matthew Jerry, Suman Datta, Arijit Raychowdhury

**Affiliations:** 1 Georgia Institute of Technology, Atlanta, GA USA; 2 University of Notre Dame, Notre Dame, IN USA

Correction to: *Scientific Reports* 10.1038/s41598-017-00825-1, published online 19 April 2017

This Article contains a typographical error. In the Results section under subheading ‘Analytical Model, Piecewise Linear System Dynamics and connection to Spectral Algorithms’,

“If the voltage *v* is normalized to *v*_*dd*_ then *p*(*s*) = *s*.”

should read:

“If the voltage *v* is normalized to *v*_*dd*_ then *p*(*s*) = *g*_*m*_*s*.”

In addition, all instances of *g*_*i*_*s* should be *g*_*m*_*s*.

