# Supplementary material for: Vertex coloring of graphs via phase dynamics of coupled oscillatory networks
Source: Sci Rep. 2017 Apr 19;7:911. doi: 10.1038/s41598-017-00825-1 (PMC5430425; doi:10.1038/s41598-017-00825-1)
Supplement: Supplementary file 1 — Supplementary Text [file 41598_2017_825_MOESM1_ESM.pdf]

Vertex coloring of graphs via phase dynamics of coupled oscillatory networks
(Supplementary Text)

Abhinav Parihar, Nikhil Shukla, Matthew Jerry, Suman Datta, Arijit Raychowdhury

Notations

- Scalars and vectors are denoted by lower case variables.
- Matrices are denoted by upper case variables.
- Single subscripts denote indices for vectors and corresponding columns for matrices.
- Double subscripts denote corresponding elements for matrices.
- General results about the asymptotic order are proved using x as the state vector. In the context of the paper, the system being described is the relaxation oscillator system and the state vector x refers to the output voltage $v(t)$.
- The state vector representing states of all oscillators is denoted by lower case s and the diagonal matrix constructed using the state vector as diagonal is denoted by upper case $\hat{S}$.

Summary

Following sections describe the proposed coupled relaxation oscillator system in detail.

- Section 1 describes the piecewise linear dynamics of a system of a coupled relaxation oscillators.
- Section 2 focusses on dynamics in the particular discharge state $s = 0$ and explains its relevance and the relationship between eigenvectors of the coefficient matrix and the asymptotic order of components of the state vector x in the discharge state $s = 0$.
- Section 3 discusses similar arguments in other states $s \neq 0$.
- Section 4 explains the reformulation of vertex coloring as vertex color-sorting.
- In section 5 we discuss the existence of a periodic cycle in the case of complete partite graphs with equal nodes in each class of the partition. The current system can provide the correct, albeit non-optimal coloring for sparse graphs.
- In section 6 we give reasons for extending such arguments to general graphs and why the system moves away from the conditions as graphs become sparser.

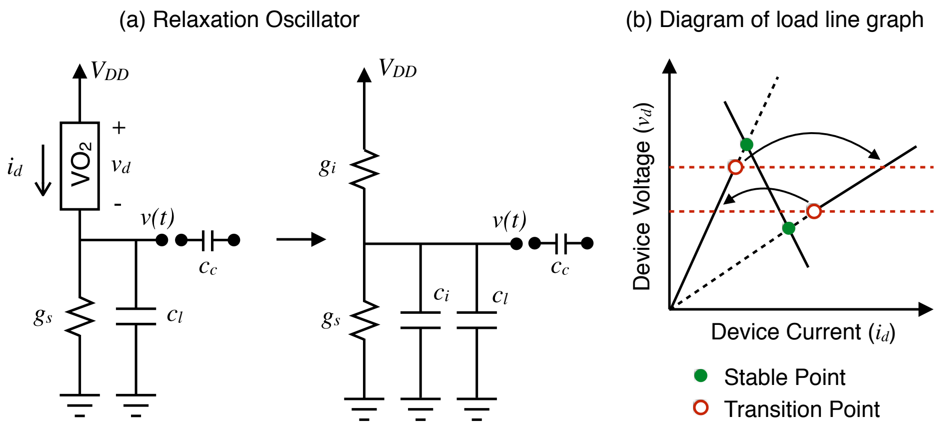

Figure 1.1: (a) A relaxation oscillator circuit and its equivalent circuit in terms of intrinsic conductance and capacitance. (b) Load line graph and I-V curve of the device showing transition points, stable points and oscillations due to hysteresis.

- Section 7 describes necessary background for the experimental implementation of such coupled relaxation oscillators using VO_2 (Vanadium Dioxide) devices.
- The Appendix contains some results useful for analyses in Section 5.

1 Dynamics of a system of coupled relaxation oscillators

We consider a system of n coupled VO_2 oscillators, where each oscillator is a series combination of a VO_2 device, and a parallel combination of a series conductance g_s and a loading capacitance c_l. The VO_2 device is an MIT (metal-insulator-transition) device which switches between a metallic state and an insulating state depending on the voltage v across it. When $v > v_h$ the device switches to a metallic state, and when $v < v_l$ the device switches to an insulating state. $v_l \neq v_h$ and there is hysteresis, i.e. system tries to retain the last state when $v_l \leq v \leq v_h$. When a VO_2 device is connected in series with a resistance of appropriate magnitude, it shows self sustained oscillations. As can be seen in figure 1.1b, because the stable points of the circuit in both the states (metallic and insulating) lie outside the region of operation, i.e. they are preceded by a transition, the system never settles to a point.

The dynamics of the coupled system with n oscillators coupled pairwise to each other using capacitances can be written as:

$$(C_i + C_c + C_l)\, v'(t) = -G(s)v(t) + H(s) \tag{1.1}$$

where s is the state of the system, $s = \{s_1, s_2, \cdots, s_n\}$, s_k being the state of kth oscillator and $v(t)$ is the vector of all the output voltages of oscillators.. C_i is the intrinsic internal capacitance matrix and C_l is the loading capacitance matrix. These are diagonal matrices with each element equal to the corresponding capacitance of the oscillator.

$$C_i = \begin{pmatrix} c_{i1} & & 0 \\ & \ddots & \\ 0 & & c_{in} \end{pmatrix}, C_l = \begin{pmatrix} c_{l1} & & 0 \\ & \ddots & \\ 0 & & c_{ln} \end{pmatrix}$$

where c_{ik} is the internal capacitance and c_{lk} is the loading capacitance of kth oscillator.

C_c is the coupling capacitance matrix

$$C_c = \begin{pmatrix} \sum & -c_{c_{12}} & \cdots & -c_{c_{1N}} \\ -c_{c_{21}} & \sum & & -c_{c_{2N}} \\ \vdots & & \ddots & \\ -c_{c_{N1}} & -c_{c_{N2}} & & \sum \end{pmatrix}$$

where $c_{c_{ij}}$ is the coupling capacitances between i^{th} and j^{th} oscillators, and $\sum$ represent the sum of rows (or columns). When all the coupling capacitances are equal to c_c, then C_c is basically the scaled Laplacian matrix L of the graph with $C_c = c_c L = c_c(D - A)$ where D is the diagonal matrix of degrees of vertices and A is the adjacency matrix of the graph. It should be noted that the loading capacitances are chosen such that $diag(C_c + C_l)$ is constant. We envision a system where the oscillators are connected in a graph which is topologically equivalent to the input graph. As such the coupling matrix is programmed by the incidence matrix of the input graph, For each row i in C_c every absent edge ij in the graph adds a loading capacitance of magnitude c_c to the ith node to maintain a constant $diag(C_c + C_l)$. This ensures equal loading effect for all the nodes and symmetric dynamics.

$G(s)$ and $H(s)$ are state dependent matrices

$$G(s) = \begin{pmatrix} g_1(s_1) & & 0 \\ & \ddots & \\ 0 & & g_N(s_2) \end{pmatrix}, H(s) = \begin{pmatrix} h_1(s_1) \\ \vdots \\ h_N(s_N) \end{pmatrix}$$

where

$$g_k(s_k) = \begin{cases} g_{ik} + g_{sk} & s_k = 1, (charging) \\ g_{sk} & s_k = 0, (discharging) \end{cases}$$

and

$$h_k(s_k) = \begin{cases} g_{ik} & s_k = 1, (charging) \\ 0 & s_k = 0, (discharging) \end{cases}$$

with g_{ik} and g_{sk} being the internal conductance and the series conductance of the kth oscillator respectively.

This can be written as:

$$v'(t) = (C_i + C_c + C_l)^{-1} \left[-G(s)v(t) + H(s)\right]$$

where voltages are normalized to V_{DD}. In rest of the text, the state vector will be represented by $x(t)$ instead of $v(t)$.

1.1 A symmetric system with identical oscillators

Let us first consider a symmetric system, i.e. equal internal capacitances (c_i), coupling capacitances (c_c), internal conductances (g_i) and series conductances (g_s). In such case, $(C_i + C_c + C_l) = (c_i I + c_c D - c_c A + C_l)$ where A is the adjacency matrix of the graph and D is the diagonal matrix of degrees of vertices. One simple choice of C_l is $C_l = c_c(nI - D)$ which makes

$$\begin{aligned} diag(C_c + C_l) &= diag(c_c D - c_c A + c_c nI - c_c D) \\ &= diag(c_c nI) \\ &= c_c n \, diag(I) \end{aligned}$$

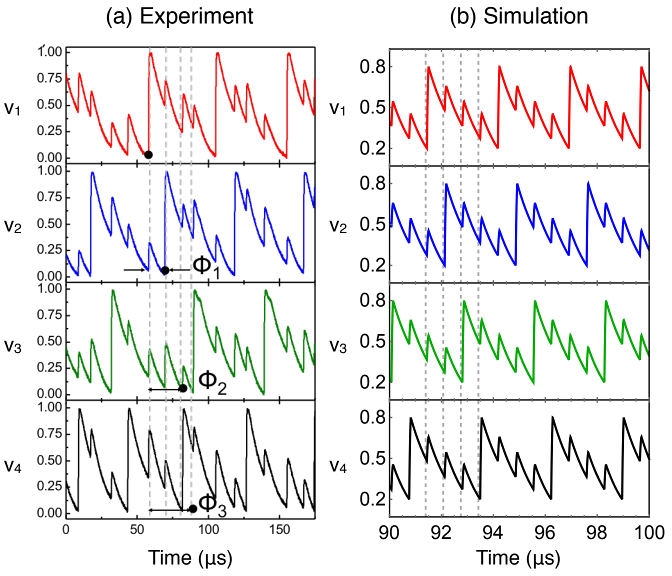

Figure 1.2: Experimental (a) and simulated (b) waveforms of a coupled relaxation oscillator circuit connected in a complete graph with 4 nodes.

which is constant. Hence the coefficient matrix becomes

$$-G(s)(c_i I - c_c A + c_c n I)^{-1} = G(s)\,(c_c A - (c_i + c_c n)I)^{-1}$$

Let us define $B = (c_c A - (c_i + c_c n)I)^{-1}$. Also let $\hat{S}$ be a diagonal matrix where $diag(\hat{S}) = s$. Then $H(s) = g_i s$ and $G(s) = g_s I + g_i \hat{S}$ where I is the identity matrix. The system of 1.1 can then be written as:

$$v'(t) = B\left(g_i \hat{S} v + g_s\,(s - v)\right) \tag{1.2}$$

We note two important features about the charging transitions: (a) charging processes are very fast compared to the period of oscillations (figure 1.2), which we also refer to as "charging spikes" and (b) Charging of one oscillator has weak (but finite) effect on the other oscillators. Hence, we study the dynamics of coupled relaxation oscillator system in terms of two distinct interacting systems - the linear dynamics in the discharging state $s = 0$, and the charging transitions.

As the charging processes are very fast, the relative phases of oscillators are same as the relative times of the charging spikes in the oscillator waveforms. This gives a good way to visualize how the relative phases of oscillators evolve with time. For all oscillators, we first note all the time instants when the charging spikes start. The time differences between consecutive charging spikes should settle to a constant value if the oscillators settle, say Δt_i for the ith oscillator. If all the oscillators synchronize to a common frequency then $\Delta t_i = \Delta t_0$ for all i. Then at any n^{th} charging spike which occur at time instant t_n, we can calculate the relative phase of an oscillator w.r.t. a hypothetical oscillator whose charging spikes occur at regular intervals of Δt_i from the start ($t = 0$) as:

$$\phi(n) = (t_n - n\Delta t_i)\frac{2\pi}{\Delta t_i}\ (mod\,2\pi)$$

When all Δt_i are equal, i.e. the oscillators synchronize, $\phi(n)$ calculates the relative phases w.r.t. a common Δt_0 for all oscillators. We plot $\phi(n)$ vs n for all oscillators in figure 1.3. What we observe is that the phases $\phi(n)$ converge

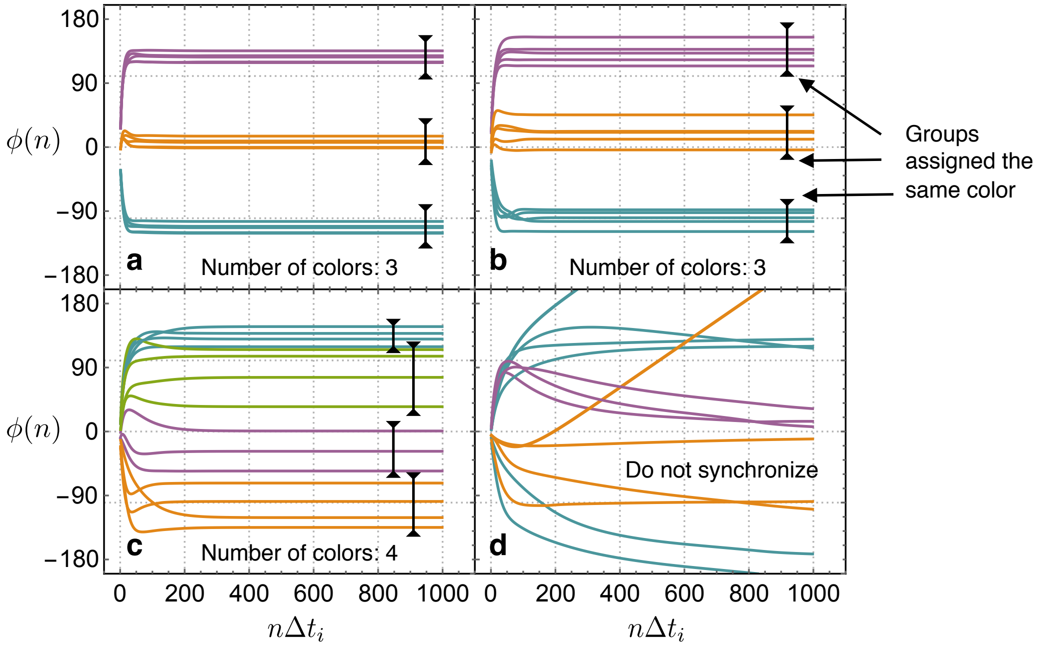

Figure 1.3: The phases $\phi(n)$ plotted against $n\Delta t_i$ for four relaxation oscillator systems for solving 3-colorable graphs with the same color partition $(5, 5, 5)$ but with different connectivities. Case (a) is the case of a complete 3-partite graph, and graphs become sparser from (a) to (d). The phase clustering degrades as graphs become sparser and for very sparse graphs (d) the oscillators do not synchronize. The number of colors detected using our algorithm is shown with each graph and the nodes which are assigned the same color are indicated.

and cluster together for dense graphs but as the graphs become sparse, which are considered harder, the the phases do not converge. In the intermediate region between dense and very sparse graphs, the phase do converge but they do not cluster together in groups. In these case our proposed algorithm and reformulation of vertex coloring is particulalry useful because it does not rely on the clustering of phases. Our algorithm does an $O(n^2)$ post-processing on the steady state order of phases and calculates a color assignment which is always correct but can have non-optimal coloring, i.e. the number of colors can be more than the chromatic number.

2 Linear dynamics in the discharge phase $s = 0$

In the state $s = 0$ where all the oscillators are in the discharging state, the system is an autonomous linear dynamical system

$$x'(t) = -g_s\left(c_i I + c_c L + C_l\right)^{-1} x(t)$$

Hence, the time evolution of this dynamical system is governed by the spectral properties of the coefficient matrix. In an identical system, the equation is

$$\begin{aligned} x'(t) &= g_s\left(c_c A - (c_i + n c_c) I\right)^{-1} x(t) \\ &= g_s B v(t) \end{aligned}$$

Let the eigenvectors of B be μ_k.

Proposition 1. *The eigenvectors of the coefficient matrix B of the identical system are the same as those of the adjacency matrix A. The eigenvalues μ_k of B are related to the eigenvalues of A as follows:*

$$\mu_k = \frac{1}{c_c\left(\lambda_k - \frac{c_i}{c_c} - n\right)}$$

Moreover, $\mu_k < 0$ for $1 \leq k \leq n$.

Proof. For any matrix M with an eigenvalue m, the eigenvectors of $M + \alpha I$ and $\beta(M + \alpha I)^{-1}$ are same as M for any scalars α and β. This can be seen as follows:

$$\begin{aligned}(M + \alpha I)\,x &= Mx + \alpha x \\ &= (m + \alpha)\,x\end{aligned}$$

And eigenvectors remain unchanged for matrix inverse. Also eigenvalues for $\beta(M + \alpha I)^{-1}$ will be $\beta/(m + a)$. Substituting appropriate values for α and β gives us the required relation between μ_k and λ_k.

Now, the Perron-Frobenius theory [1]implies that largest eigenvalue of A is less than or equal to the maximum row sum which is less than n, i.e.

$$\lambda_{max} \leq r_{max} < n$$

Hence, $\left(\lambda_k - \frac{c_i}{c_c} - n\right) < 0$ for all k which implies that $\mu_k < 0$ for all k. □

2.1 Asymptotic trajectories and asymptotic order of components of the state vector in a linear dynamical system

In a linear dynamical system with the state variable $x(t)$, the order of components of $x(t)$ define a permutation at any time instant t. In state $S = 0$, the linear dynamical system is

$$x'(t) = Bx(t)$$

where B is real, symmetric and the initial state of the system $x(0) = x_0$.

Geometry of permutation regions

For any ordering P of components $x_{i1} > x_{i2} > ... > x_{in}$, the region that corresponds to this ordering is given by

$$\mathcal{R}_{\mathcal{P}}(P) = \bigcap_{m=i}^{n} \left(x_{im} > x_{i(m+1)}\right) \tag{2.1}$$

$\mathcal{R}_{\mathcal{P}}(P)$ is a pair of n-dimensional simplexes with one vertex as the origin and are mirror images of each other about the origin. As such, any line that passes through the origin either passes through both of them, or none.

Asymptotic direction of trajectories

In a linear dynamical system, the asymptotic order of components is hence governed by the asympotic direction in which the system state converges to.

Proposition 2. *In the linear dynamical system $x'(t) = Bx(t)$, where the coefficient matrix B is real, symmetric and full-rank, the system trajectory always converges asymptotically to a particular direction. Moreover, if the asymptotic direction is given by $d(x_0, B)$ where $x(0) = x_0$, then $d(x_0, B)$ lies in the eigenspace of B with the largest eigenvalue (including the sign) almost everywhere, i.e. when the system starts from anywhere except on a set of measure 0.*

Proof. Let $x(t, x_0)$ be the solution of the dynamical system when the initial starting state $x(0) = x_0$. As the fixed point is 0, the asymptotic direction $d(x_0, B)$ to which the system state converges can be written as

$$\begin{aligned} d(x_0, B) &= \lim_{t\to\infty} \frac{x(t)}{||x(t)||} \\ &= \lim_{t\to\infty} \frac{e^{Bt}x_0}{e^{\lambda(x_0)t}} \end{aligned}$$

where $\lambda(x_0)$ is the Lypunov exponent of the trajectory starting from x_0. As B is real and symmetric, all its eigenvalues are real and the matrix is diagonalizable. Let $B = Q\Lambda Q^T$, where Λ is the diagonal matrix with of all eigenvalues. Then

$$d(x_0, B) = Q\left(\lim_{t\to\infty} \frac{e^{\Lambda t}x_0}{e^{\lambda(x_0)t}}\right) Q^T x_0$$

Let $\lambda_1 > \lambda_2 > ... > \lambda_l$ be the l distinct eigenvalues of B, and let E_k, $1 \le k \le l$ be the corresponding eigenspaces. Now, $\lambda(x_0) = \lambda_1$ for $x_0 \in \bigoplus_{k=1}^{l} E_k \backslash \bigoplus_{k=1}^{l-1} E_k$. This means $\lambda(x_0) = \lambda_1$ almost everywhere, i.e. everywhere except on a set of measure 0. Hence

$$\begin{aligned} d(x_0, B) &= Q\begin{pmatrix} 1 & 0 & 0 & \cdots & 0 \\ 0 & 1 & 0 & & \\ 0 & 0 & \ddots & & \\ \vdots & & & 0 & \\ 0 & & & & \ddots \end{pmatrix} Q^T x_0 \\ &= \left(q_{1a}q_{1a}^T + q_{1b}q_{1b}^T + ...\right) x_0 \\ &= P_{E_1} x_0 \end{aligned}$$

Here, the diagonal elements of the middle matrix are ones only for the rows corresponding to the eigenvector λ_1, and $q_{1a}, q_{1b}, ...$ are orthogonal vectors that span E_1. Hence $d(x_0, B) \in E_1$ almost everywhere. In case the largest eigenvalue λ_1 of B has multiplicity 1, $d(x_0, B)$ is simply q_1 a.e. □

Asymptotic order of components

The asymptotic order of components of $x(t)$ is determined by the permutation region in which $d(x_0, B)$ lie. Let $T(v)$ denote the order of components of vector v, then $T(d(x_0, B)) = T(P_{E_1}x_0)$ is the asymptotic order of components of $x(t)$. The asymptotic order becomes a little more complex when $d(x_0, B)$ lies at the boundary of two or more permutation regions, i.e. some of the components of $d(x_0, B)$ are equal. In such cases, $T(d(x_0, B))$ is only a partial order as determined by $d(x_0, B)$. $T(d(x_0, B))$ can be extended to a total order by the asymptotic direction of the system in the remaining space $E_2 \oplus E_3 \oplus ... \oplus E_l$. Let us denote this by $d(x_0 \backslash E_1)$. Also, let P_{E_1} be the projection matrix on E_1, then

$$d(x_0, B\backslash E_1) = \lim_{t\to\infty} \frac{(I - P_{E_1})\, x(t)}{\left\|(I - P_{E_1})\, x(t)\right\|}$$

Now, $d(x_0, B\backslash E_1) \perp d(x_0, B)$. When $d(x_0, B)$ is at the boundary of some permutation regions, the disambiguation among these regions, i.e. ordering among the components which are equal, is done by $d(x_0, B\backslash E_1)$ as it is perpendicular to $d(x_0, B)$. Hence, the asymptotic order is determined by both $d(x_0, B)$ and $d(x_0, B\backslash E_1)$. If $d(x_0, B\backslash E_1)$ lie at the boundary of some other permutation regions, then the argument can be extended in a similar way and the asymptotic order of components is determined by $d(x_0, B)$, $d(x_0, B\backslash E_1)$ and $d(x_0, B\backslash E_1 \oplus E_2)$ together, and so on.

The extension of the partial order $T(d(x_0, B))$ using $T(d(x_0, B\backslash E_1))$ is similar to the ordinal sum $T(d(x_0, B)) \oplus T(d(x_0, B\backslash E_1))$ but a preferential one, i.e. the orders determined by $T(d(x_0, B))$ are preferred over those determined in $T(d(x_0, B\backslash E_1))$. Let us denote this operation by the binary operator $\oplus'$ which acts on an ordered pair of two partial orders and gives another partial or total order.

The range of $(I - P_{E_1})$ is $E_2 \oplus E_3 \oplus ... \oplus E_l$. The dynamics that govern the time evolution of $(I - P_{E_1})\, x(t)$ in the space $E_2 \oplus E_3 \oplus ... \oplus E_l$ is simply determined by the eigenvectors and eigenvalues corresponding to $E_2, E_3, ..., E_l$. Hence from 2, $d(x_0, B\backslash E_1) \in E_2$. Specifically,

$$d(x_0, B\backslash E_1) = \left(q_{2a}q_{2a}^T + q_{2b}q_{2b}^T + ...\right) x_0$$

where $q_{2a}, q_{2b}, ...$ are the eigenvectors corresponding to λ_2. Extending the argument, we have $d(x_0 \backslash E_1 \oplus E_2) \in E_3$ and so on. Hence, we have the following:

Proposition 3. *The asymptotic order of components of $x(t)$ in the linear dynamical system $x'(t) = Bx(t)$, where the coefficient matrix B is real, symmetric and full-rank, is determined by $T(d(x_0, B))$. In case $d(x_0, B)$ lies on the boundary of some permutation regions then $T(d(x_0, B))$ is a partial order which can be extended to a total order as $T(d(x_0, B)) \oplus' T(d(x_0, B\backslash E_1))$. And in case $d(x_0, B\backslash E_1)$ lies at some boundary then the asymptotic order is determined as $T(d(x_0, B)) \oplus' T(d(x_0, B\backslash E_1)) \oplus' T(d(x_0, B\backslash E_1 \oplus E_2))$. Moreover,*

$$\begin{aligned} d(x_0, B) &= \left(q_{1a}q_{1a}^T + q_{1b}q_{1b}^T + ...\right) x_0 = P_{E_1}x_0 \in E_1 \\ d(x_0, B\backslash E_1) &= \left(q_{2a}q_{2a}^T + q_{2b}q_{2b}^T + ...\right) x_0 = P_{E_2}x_0 \in E_2 \\ d(x_0, B\backslash E_1 \oplus E_2) &= \left(q_{3a}q_{3a}^T + q_{3b}q_{3b}^T + ...\right) x_0 = P_{E_3}x_0 \in E_3 \end{aligned}$$

and so on. Hence, the asymptotic order of components is determined as

$$Q_0(x_0) = T(P_{E_1}x_0) \oplus' T(P_{E_2}x_0) \oplus' T(P_{E_3}x_0) \dots$$

3 Linear dynamics in the charging states $s \neq 0$

When $s \neq 0$ the system is a linear dynamical system, but the fixed point is not 0. The identical system in a charging state s can be described as

$$\begin{aligned} v'(t) &= B\left[G(s)v(t) - H(s)\right] \\ &= BG(s)\left(v(t) - G(s)^{-1}H(s)\right) \end{aligned}$$

The fixed point of the system in a state s is

$$G(s)^{-1}H(s) = \frac{g_i}{g_s + g_i} s$$

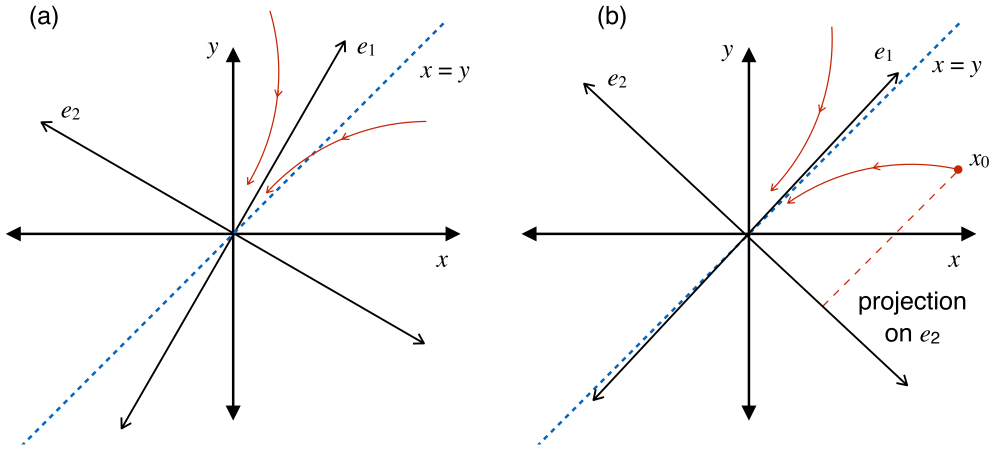

Figure 2.1: Representation of flows in a two dimensional linear dynamical system where both eigenvalues are negative and $|\lambda_2| > |\lambda_1|$. (a) The system trajectory approaches the direction of e_1 with time and hence the order of components, i.e. the order of x and y coordinates is determined by e_1. (b) When e_1 lies close to the $x = y$ line, the order depends on which side x_0 lies w.r.t. e_1 which is given by the projection of x_0 on e_2.

and the coefficient matrix for the linear flow is

$$(c_c A - (c_i + c_c n)I)^{-1}\, G(s) = BG(s)$$

where $B = (c_c A - (c_i + c_c n)I)^{-1}$ as before (section §2). When $g \gg g_s$, i.e. the chargings are much faster than the dischargings, the fixed points of the system are close to s which are the corners of the unit cube in n dimensions. Following the arguments as in section §2, even in this case the system trajectory will converge to an asymptotic direction. The asymptotic ordering of components would depend on first the fixed point, and in case the fixed point has equal components then it would also depend on the asymptotic direction of trajectory. This is explained as:

Proposition 4. *In the linear dynamical system of the charging states $x'(t) = BG(s)\,(x(t) - p)$, where $p = \frac{g}{g_s+g} s$ is the fixed point and the coefficient matrix B is real, symmetric and full-rank, the asymptotic permutation of the components will be same as the permutation of components of the fixed points, i.e. $T(p)$. In case the fixed point p lies at (or close) to the boundary of some permutation regions, i.e. some components of p are equal, the disambiguation of ordering among these components can be done considering the linear dynamics of $x'(t) = Bx(t)$ with fixed point shifted to 0, and following Propositions 3. Hence, the asymptotic order of components is given by*

$$\begin{aligned} Q_s(x_0) &= T(p) \oplus' T(P_{sE_1} x_0) \oplus' T(P_{sE_2} x_0) \oplus' \ldots \\ &= T(s) \oplus' T(P_{sE_1} x_0) \oplus' T(P_{sE_2} x_0) \oplus' \ldots \end{aligned}$$

where $P_{sE_1}, P_{sE_2}, \ldots$ are the projections on the eigenspaces of $BG(s)$.

In case the matrix B in the equation $x'(t) = BG(s)\,(x(t) - p)$ is not full rank, the system trajectory does not converge to the point p. If N is the null space of the matrix B and P_N is the projection on the null space N, then the convergence limit point for the trajectory starting from x_0 is is $p + P_N x_0$. Also, N is also the null space for $BG(s)$ for all s. Hence, Proposition 4 can be modified for matrices B which are not full-rank as follows

Proposition 5. *In the linear dynamical system as described in Proposition 4, but where B is not full rank, the asymp-*

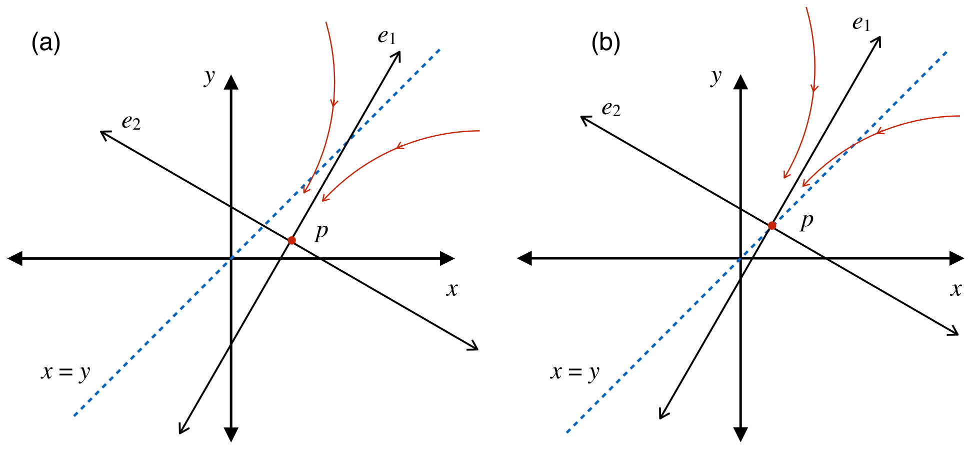

Figure 3.1: (a) When the fixed point in a two dimensional linear dynamical system is not 0 then the asymptotic order of the components is determined by the fixed point p. (b) If the fixed point lies on the $x = y$ line, which is a boundary of permutations regions, then the disambiguation is done using the eigenvectors.

totic order of components is given by

$$Q_s(x_0) = T\left(\frac{g_i}{g_i + g_s}s + P_{sN}x_0\right) \oplus' T(P_{sE_1}x_0) \oplus' T(P_{sE_2}x_0) \oplus' \ldots$$

where P_{sN} is the projection matrix on the null space of $BG(s)$.

When x_0 is close to the eigenspaces, i.e. magnitude of $P_{sN}x_0$ is very small, the additive term of $P_N x_0$ in the first term does not change the order determined by s. Formally, when $\max\{(P_N x_0)_i\} < \frac{g_s}{g_i+g_s}$

$$T\left(\frac{g_i}{g_i + g_s}s + P_N x_0\right) = T(s) \oplus' T(P_N x_0)$$

and hence,

$$Q_s(x_0) = T(s) \oplus' T(P_{sN}x_0) \oplus' T(P_{sE_1}x_0) \oplus' T(P_{sE_2}x_0) \oplus' \ldots \tag{3.1}$$

3.1 Approximation by instantaneous charging

If the chargings are very fast, i.e. $\frac{g_s}{g_i} \to 0$, we can approximate the chargings by an instantaneous change in the state from x to $x + \Delta x$ by linearizing the system at the time instant when the state changes from $s = 0$ to the charging state. Let $\hat{S}$ denote a diagonal matrix such that $diag(\hat{S}) = s$ where s is the state vector. When $s \neq 0$ we have from (1.2)

$$\begin{aligned} x'(t) &= B\left(g_i\hat{S}x + g_s(s - x)\right) \\ &= Bg_i\left(\hat{S}x + \frac{g_s}{g_i}(s - x)\right) \\ &\simeq g_i B\hat{S}x \end{aligned}$$

If the kth node charges then $\hat{S}x = v_l e_k$ where e_k is $k - axis$ vector whose all components are 0 expect the kth which

is 1. If the kth node charges completely from v_l to v_h without any state transition in between, we have

$$\begin{aligned} (\Delta x)_k &= dv \\ \implies (x')_k \Delta t &= dv \\ \implies \Delta t &= \frac{dv}{(g_i B \hat{S} x)_k} \\ &= \frac{dv}{g_i v_l e_k^T B e_k} \\ &= \frac{dv}{g_i v_l B_{kk}} \end{aligned}$$

Therefore,

$$\begin{aligned} \Delta x &= x' \Delta t \\ &= dv \frac{v_l g_i B e_k}{v_l g_i B_{kk}} \\ &= \frac{dv}{B_{kk}} B e_k \end{aligned}$$

which is just a scaled column vector of B. We have the following:

Proposition 6. *In the dynamical system of (1.2), when* $s \neq 0$ *and only a single node charges, the chargings can be approximated by linearizing the system. If the transition occurs from* x *to* $x + \Delta x$ *then* Δx *is given by:*

$$\Delta x = \frac{dv}{B_{kk}} B e_k$$

Remark 1. An important point to note here is that this change is independent of x.

4 Vertex Color-Sorting

As can be seen in the system equation of the capacitively coupled oscillators, the discharge phase (where all oscillators are discharging) is a simple linear differential equation with $H(s) = 0$. The matrix $C - C_C$ is just the Laplacian matrix of the graph of the oscillators and the system dynamics is governed by simply the eigenspectrum of the of the Laplacian matrix of the graph. As such, there are interesting connections between spectral algorithms for graph coloring and the coupled relaxation oscillator circuit.

Definition 1. (k-Color-Sorting) An ordering $u = \{u_i\}$, $i \in [1, n]$ of the n nodes of a graph is a proper k-Color-Sorting if there exists a proper k-Coloring $\{c_i\}$, $i \in [1, n]$, where c_i is the color assigned to the ith node such that all nodes with the same color appear together in u, i.e. for any nodes i, j, k with $u_i < u_k < u_j$, $c_i = c_j \implies c_i = c_k = c_j$. This can be extended to a cyclic ordering where the nodes with the same color appear together.

Lemma 1. *For a graph with n nodes, adjacency matrix A and chromatic number* χ_A*:*

1. Any ordering of nodes S is a proper k-Color-Sorting for some k such that $\chi_A \leq k \leq n$.

2. Let $B(M)$ be the minimum number of diagonal blocks which are identically $'0'$ and which cover the complete diagonal of the matrix M. The minimum k for which S is a proper k-Color-Sorting is $B(PAP^T)$. If S is a proper

k-Color-Sorting and P its permutation matrix, then

$$\chi_A \leq B(PAP^T) \leq k$$

Proof. Any ordering S is a proper n-Color-Sorting, and if S is a proper k color sorting then minimum number of colors can be χ_A.

If P is the permutation matrix of an ordering u, then PAP^T is the adjacency matrix of a graph with the ordering of nodes changed to u. If u is a proper k-Color-Sorting then, PAP^T will have at least k number of $'0'$ diagonal blocks, one corresponding to each color group, hence, $B(PAP^T) \leq k$. Also, the diagonal blocks which are $'0'$ also determine a valid coloring of the graph and hence $B(PAP^T) \geq \chi_A$. □

Proposition 7. *For a k-chromatic graph, k-Color-Sorting is NP hard. Moreover, finding the chromatic number χ_A of a graph with adjacency matrix A and the proper χ_A-Coloring is equivalent to the following optimization problem:*

$$min\, B(PAP^T), \quad P \in all\, permutations\, of\, nodes$$

where the solution P is a proper χ_A-Color-Sorting, $\chi_A = min\{B(PAP^T)\}$.

Proof. Computing $B(PAP^T)$ is a $O(n^2)$ problem, n being the number of nodes because there are n^2 elements in PAP^T. And for a k-chromatic graph, $\chi_A = B(PAP^T) = k$ where P is a proper k-Color-Sorting. Hence, χ_A can be computed in $O(n^2)$ if a proper k-Color-Sorting P can be found.

Also, for any permutation P, $B(PAP^T) \geq \chi_A$ as stated above, where equality holds only when P is a proper χ_A-Color-Sorting. Hence, finding chromatic number is equivalent to the stated optimization problem. Also, once a proper χ_A-Color-Sorting is known, the $'0'$ diagonal blocks also determine the proper χ_A-Coloring. □

5 Cycles in the prototypical case: complete graphs with equal nodes in each class

Using the results in the previous sections, we can understand why a cycle would exist in the prototypical case of a complete graph when the number of nodes in each class is equal.

Proposition 8. *The following three conditions when satisfied result in the existence of a cycle and helps us understand why the possibility of it reduces as graphs become sparser, and hence harder.*

1. ***Attractor:*** *The system in state $s = 0$ tries to order the components of the state vector in the correct vertex color-sorting. Hence, if the system starts from a state x_0 whose order of components is same as the final asymptotic order, i.e. $T(x_0) = Q_0(x_0)$, then with time $T(x(t))$ remains constant.*

2. ***Ordering:*** *The charging spikes just change the order of components of x by a circular permutation. If the kth oscillator charges from v_l to v_h then the order of all other components remains same.*

3. ***Sustaining the cycle:*** *If condition 2 is true then the charging transitions cycle the order of x_0 to all the circular permutations. For a cycle to exist, the state $s = 0$ should not only preserve the order of x_0 when $T(x_0) = Q_0(x_0)$ but it should also have lower tendency to change the order when $T(x_0)$ is any circular permutation of $Q_0(x_0)$.*

Why these conditions hold in the prototypical case of complete graph with equal number of nodes in each color class can be seen as follows.

Explanation for condition 1: The adjacency matrix A in the prototypical case is a low rank matrix with the rank equal to the number of colors, i.e. if it is a k-partite graph then rank is n. The adjacency matrix is a block matrix with equal sized k^2 blocks and the diagonal blocks are 0 and the non-diagonal blocks are 1. One eigenvector of the matrix A is the constant vector $[1, 1, 1, ...]$ which is the diagonal of the n-dimensional cube $[v_l, v_h]^n$ and also lies at the intersection of all the simplexes of the permutation regions (equation 2.1) and does not affect the asymptotic order of components of x. Hence all the other eigenvectors decide the asymptotic order and lie in the non-positive quadrants. The eigenvectors of B with least negative eigenvalues (which are the eigenvectors of A with most negative eigenvalues) have components which are equal on each color class (Appendix A.1) and hence should direct the system towards a correct vertex color-sorting in state $s = 0$. We also know that all the eigenvalues of the coefficient matrix in the state $s = 0$ are negative, and hence, if the system starts with the correct order of components, i.e. $T(x_0) = Q_0(x_0)$ then the system state x will continue to lie in the same permutation region with time.

Explanation for condition 2: Assuming very fast charging and using the instantaneous charging approximation, we see from Proposition 6 that the state transition Δx is in the direction of the kth column vector of B when the kth node charges. As shown in appendices A.2 and A.3, in case of weak coupling, i.e. $c_i \gg c_c$ the kth column vector is constant for all non-charging components and hence Δx does not change the order of the non-charging components. The variation in the non-charging components of Δx is inversely propotional to $n + m$ and hence with larger n and m the charging transition $x \to x + \Delta x$ tries to preserve the order of non-charging components more (Appendix A.3). As shown in figure 5.1 the effect of charging transitions can be seen as small kinks in the waveforms of non-charging components. The magnitude of these kinks is negligible for weak coupling (a), and is clearly visible for stronger coupling (c). Even though the charging transitions affect the non-charging components in the case of a stronger coupling, the order of non-charging components is not disturbed, i.e. the change in all the non-charging components is almost the same (Appendix A.3).

Explanation for condition 3: If the system state x is close to the eigenspace of B with least negative eigenvalue, say E_1, then x has components which are close for the same color class (Appendix A.1) and components of different color classes will have more separation between them by comparison. If the components of x are ordered in increasing order then it will have a pattern $\{x_{a_1}, x_{a_2}, ..., x_{b_1}, x_{b_2}, ..., x_{c_1}, x_{c_2}, ...\}$, where a_i are the indices for one color class, b_i for another etc. If the order among the color classes is changed, say $\{x_{b_1}, x_{b_2}, ..., x_{a_1}, x_{a_2}, ..., x_{c_1}, x_{c_2}, ...\}$ even then x will be close to the eigenspace E_1 because of the multiplicity of the least negative eigenvalue (Appendix A.1). The charging transitions of nodes of the same color class will occur consecutively with little time durations between them. This little time does not allow the system state $s = 0$ which occurs between these transitions to change the order. When all nodes of one particular class have undergone charging processes, the system state x again comes close to the eigenspace E_1 because the components of x belonging to the same color class are again close to each other. Hence, the state $s = 0$ does not disurb this order as well. The cycle repeats with very fast consecutive charging processes of the next color class. This also gives rise to clustering of the phases of nodes w.r.t. their color classes.

6 Cycles in the general case

Adjacency matrices of non-simple graphs can be considered as perturbations to the prototypical cases of complete graphs, and using perturbation theory of matrices we can say that the eigenvectors of perturbed matrices are rotations of the original eigenvectors [2], where the extent of rotation depend on the amount of perturbation. Hence, even in non-simple cases, the eigenvectors with most negative eigenvalues of the adjacency matrix will tend to have compo-

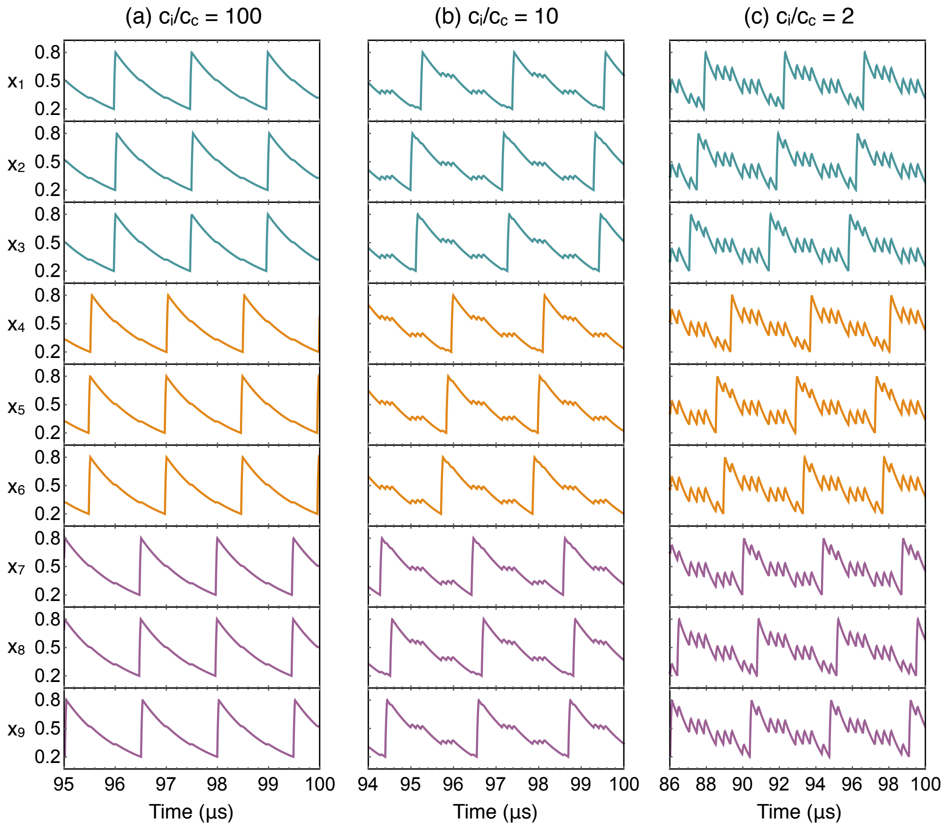

Figure 5.1: Simulation waveforms of a coupled relaxation oscillator circuit connected in a complete 3-partite graph with 3 nodes in each color class for different c_i/c_c values (a) 100, (b) 10, and (c) 2. As can be seen, the charging transitions do not affect the non-charging components of the state vector $x$in case of weak coupling (a). In case of stronger coupling (c), even though the charging transitions affect the non-charging components (seen as small kinks in the waveforms), the order of non-charging components is undisturbed as discussed in Appendix A.3.

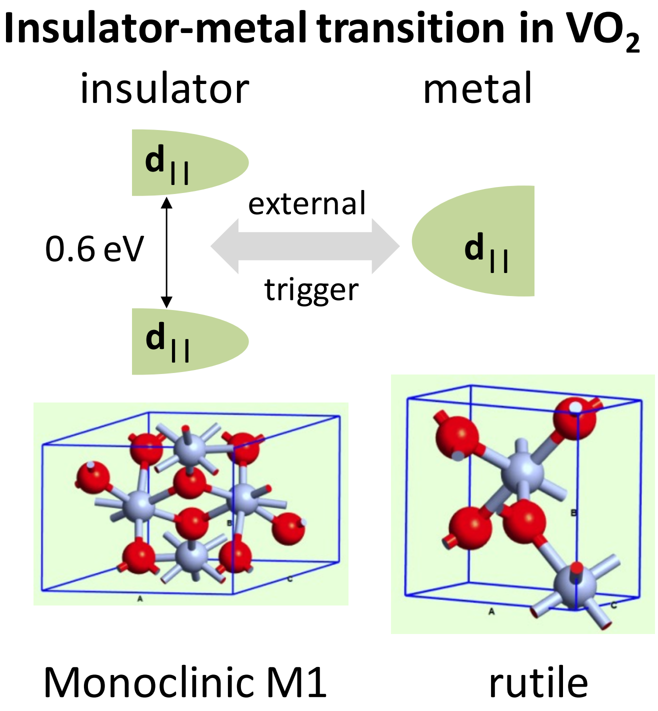

Figure 7.1: Insulator-metal transition in VO_2 showing phase change

nents which are close to each other within the same color class and away from those of different color classes. This property has been explored with mathematical detail in works related to spectral algorithms for graph coloring [3, 4, 5]. When viewed from the perspective of a coupled relaxation oscillator system of (1.2), the above mentioned property of eigenvectors of the adjacency matrix A with most negative eigenvalues will be shared by the eigenvectors of B with the least negative eigenvalues because of Proposition 1. As shown above, the asymptotic order of components of the system state in the discharge phase $s = 0$ of coupled relaxation oscillator systems depend on the least negative eigenvalues of B. Hence, the relaxation oscillator systems in state $s = 0$ is expected to direct the system towards correct vertex color sorting, which satisfies condition 1 of Proposition 8. Conditions 2 and 3 also depend on eigenvectors and hence similar arguments of matrix perturbation can be applied.

7 Prototypical experiments and validation

Vanadium dioxide (VO_2) is a prototypical insulator-metal transition material system with strong electron-electron and electron-phonon interactions that has been the subject of intense fundamental and applied research . The above room temperature phase transition (transition temperature = 340 K) in VO_2 has an electronic component characterized by an abrupt change in resistivity (and carrier concentration) up to five orders in magnitude; the large increase in carrier concentration can be attributed to collapse of the 0.6 eV band gap (optically measured) across the insulator-to-metal transition. Further, the phase transition also has a structural component wherein the crystal structure evolves from the monoclinic M1 phase with dimerized vanadium atoms in the low-temperature insulating state to rutile crystal structure in the high-temperature metallic phase.

Despite intense research efforts, the origin of the phase transition in VO_2 has been a subject of debate with competing theories suggesting that the driving force behind the transition could be Mott or Peierl's physics as well as

a weighted combination of both the mechanisms. Further, the electrically induced phase transition is VO_2 which is relevant to electronic VO_2 devices like the relaxation oscillators discussed here, is debated to be carrier density driven or of electro-thermal nature.

With respect to the relaxation oscillators discussed here, the unknown nature of origin of the electrically induced phase transition in VO_2 entails that the critical voltage (V_h, V_l in figure of main text)/ current cannot be quantitatively predicted even though empirically measurements indicate that the typical critical electric field values are in the 20-60 kV/cm range. However, we emphasize that knowing V_l and V_h, the oscillators can de designed in a deterministic manner. The details of experiments, experimental conditions and the theory connecting these experiments with linear dynamical systems for the case of a single and a coupled pair of oscillators can be found in the authors' earlier publications [6].

Following table shows the comparison of VO2 oscillators with the other promising oscillator technologies that are being developed.

	**This Work**	**TaOx [7]**	**HfTaOx and GeTe6 [8]**	**Phase Change [9]**
**Power** (μW)	12	<100	<50	<700
**Area** (μm^2)	0.89	0.5	0.5	-
**Max. Freq.** (MHz)	9	500	350	<10

Appendix

A The coefficient matrix in prototypical case

In this section we give an analytical treatment of the structure of the coefficient matrix and its eigen spectrum in the prototypical case. We consider the prototypical case where the graph is complete and the number of nodes in each color class is equal. When n identical oscillators with internal capacitances c_i are connected in a k-partite graph, and the coupling is purely capacitive with same coupling capacitances c_c used for all pairs, then the system evolution is described as in equation 1.2. In the simple case when each partition has equal number of nodes $m = n/k$, then more can be said about the coefficient matrix $B = (c_i I - c_c A + c_c nI)^{-1}$. Let $F = (c_i I - c_c A + c_c nI)^{-1}$ so that $B = F^{-1}$. Then F can be written as a repeated partitioned matrix as

$$F = U \otimes G + V \otimes E$$

where $\otimes$ is the kronecker product of matrices, U and V are $k \times k$ matrices, G and E are $m \times m$ matrices, and the matrices are given by

$$\begin{aligned} U &= c_{ic} I_k \\ G &= I_m \\ V &= I_k - J_k \\ E &= c_c J_m \end{aligned}$$

with I_m being the $m \times m$ identity matrix, J_m the $m \times m$ matrix with all ones, and $c_{ic} = (c_i + nc_c)$.

A.1 Eigenvectors of B in prototypical case

For n nodes and k color classes, let U be a $n \times m$ matrix where each column vector corresponds to one color class where the components of that particular class are k/n and rest are 0. As such, $U^T AU$ is a $k \times k$ matrix with each entry equal to the average of entries of the corresponding block in A. In the simple case of complete graph with equal number of nodes in each class, $U^T AU = J - I$ where J is a square matrix of all ones and I is the identity matrix. If x is an eigenvector of $U^T AU$ then

$$\begin{aligned} U^T AUx &= \lambda x \\ UU^T A(Ux) &= \lambda(Ux) \end{aligned}$$

Now $UU^T A$ is just the scaled version of A and hence,

$$\alpha A(Ux) = \lambda(Ux)$$

Therefore if x is an eigenvector of $U^T AU$ then Ux is an eigenvector of A. Also the number of non-zero eigenvalues of A are k which is equal to the rank of $U^T AU$ which is full-rank. Hence all the eigenvectors of A can be described using the eigenvectors of $U^T AU$ and they have equal components in a single color class. $J - I$ has an eigenvalue -1 with multiplicity $n - 1$, and an eigenvalue $n - 1$, and so does A. Now the eigenvectors of B with the least negative eigenvalues are same as that of A with most negative eigenvalues (Proposition 1). Hence, the eigenvalues of B with least negative eigenvalues are constant on each color class.

A.2 Structure of the inverse of F in prototypical case

Proposition. *If $F = (c_i I - c_c A + c_c nI)$ is the coefficient matrix of the network, then $B = F^{-1}$ has the same partitioned form as F. More precisely, $B = F^{-1}$ can be written as*

$$F^{-1} = \frac{1}{c_{ic}} \left(\frac{1}{c_{ic}} U \otimes G + D \otimes E \right)$$

where U, G and E are the same matrices that describe F, c_{ic} is as defined above, and D is a $k \times k$ matrix given by

$$D = \frac{1}{c_i + (n+m)c_c} (\beta J_k - I_k)$$

and

$$\beta = \frac{c_i + nc_c}{c_i + mc_c}$$

Proof. As described above, $F = U \otimes G + V \otimes E$. Here G is a identity matrix and E is a rank 1 matrix. Hence, as shown in [10], the inverse for F can be calculated as

$$F^{-1} = U^{-1} \otimes G - [U + (\operatorname{tr} E) V]^{-1} VU^{-1} \otimes E$$

Now,

$$\begin{aligned}
\operatorname{tr} E &= mc_c \\
U^{-1} &= \frac{1}{c_{ic}} I_k \\
VU^{-1} &= \frac{1}{c_{ic}} (I_k - J_k) \\
[U + (\operatorname{tr} E) V]^{-1} &= [U + mc_c V]^{-1} \\
&= [(c_i + (n+m)c_c) I_k - mc_c J_k]^{-1} \\
&:= [P - Q]^{-1}
\end{aligned}$$

As Q is a rank 1 matrix, we can use another result from [10]:

$$\begin{aligned}
[U + (\operatorname{tr} E) V]^{-1} &= [P - Q]^{-1} \\
&= P^{-1} + \frac{1}{1 - \operatorname{tr} QP^{-1}} P^{-1} Q P^{-1} \\
&= \frac{1}{c_i + (n+m)c_c} I_k + \frac{1}{1 - \frac{nc_c}{c_i + (n+m)c_c}} \frac{1}{(c_i + (n+m)c_c)^2} mc_c J_k \\
&= \frac{1}{c_i + (n+m)c_c} \left(I_k + \frac{mc_c}{c_i + mc_c} J_k \right)
\end{aligned}$$

Combining the parts, and noting that $J_k^2 = kJ_k$, we get

$$\begin{aligned}
[U + (\operatorname{tr} E) V]^{-1} VU^{-1} &= \frac{1}{c_i + (n+m)c_c} \left(I_k + \frac{mc_c}{c_i + mc_c} J_k \right) \frac{1}{c_{ic}} (I_k - J_k) \\
&= \frac{1}{c_{ic} (c_i + (n+m)c_c)} (I_k - \beta J_k)
\end{aligned}$$

where,

$$\beta = \frac{c_i + nc_c}{c_i + mc_c}$$

Finally,

$$F^{-1} = \frac{1}{c_{ic}} \left[I_k \otimes I_m + \frac{1}{c_i + (n+m)c_c} (\beta J_k - I_k) \otimes c_c J_m \right]$$

and hence,

$$B = F^{-1} = \frac{1}{c_{ic}} \left(\frac{1}{c_{ic}} U \otimes G + D \otimes E \right) \tag{A.1}$$

□

A.3 Column vector of B in prototypical case

Using equation A.1 we can deduce properties of the column vector of B.

Proposition. *Let B_k be the kth column vector of B and B_{kl} be the $(k,l)^{th}$ element of B. For the components of B_k there are only 3 kinds of values.*

1. *For the kth component,*

$$B_{kk} = \frac{1}{c_{ic}} (1 + \alpha(\beta - 1))$$

2. *For all other components in the same class as the kth component, i.e. when k^{th} and l^{th} node are in the same color class*

$$B_{kl} = \frac{1}{c_{ic}}\alpha(\beta - 1)$$

3. *For all other components of B_k which are not in the same partition/color class as the kth node, i.e. when k^{th} and j^{th} node are not in the same class*

$$B_{kj} = \frac{1}{c_{ic}}\alpha\beta$$

where

$$\alpha = \frac{c_c}{c_i + (n+m)c_c}$$

4. *The difference between B_{kl} and B_{kj} w.r.t. B_{kk} is given by:*

$$\frac{B_{kj} - B_{kl}}{B_{kk}} = \frac{1}{r + n + m + \frac{n-m}{r+m}}$$

where $r = c_i/c_c$. As can be seen, this difference can be made very small by weak coupling, i.e. $c_c \ll c_i$, but more importantly for increasing n and m this difference reduces

References

[1] Roger A. Horn and Charles R. Johnson. *Matrix Analysis*. Cambridge University Press, October 2012.

[2] Chandler Davis. The rotation of eigenvectors by a perturbation. 6(2):159–173.

[3] N. Alon and N. Kahale. A spectral technique for coloring random 3-colorable graphs. 26(6):1733–1748.

[4] B. Aspvall and J. Gilbert. Graph coloring using eigenvalue decomposition. 5(4):526–538.

[5] F. McSherry. Spectral partitioning of random graphs. In *42nd IEEE Symposium on Foundations of Computer Science, 2001. Proceedings*, pages 529–537.

[6] Abhinav Parihar, Nikhil Shukla, Suman Datta, and Arijit Raychowdhury. Synchronization of pairwise-coupled, identical, relaxation oscillators based on metal-insulator phase transition devices: A model study. *Journal of Applied Physics*, 117(5):054902, February 2015.

[7] Matthew D Pickett and R Stanley Williams. Sub-100 fj and sub-nanosecond thermally driven threshold switching in niobium oxide crosspoint nanodevices. *Nanotechnology*, 23(21):215202, 2012.

[8] AA Sharma, Y Kesim, M Shulaker, C Kuo, C Augustine, HS-P Wong, S Mitra, M Skowronski, JA Bain, and JA Weldon. Low-power, high-performance s-ndr oscillators for stereo (3d) vision using directly-coupled oscillator networks. In *VLSI Technology, 2016 IEEE Symposium on*, pages 1–2. IEEE, 2016.

[9] Frank C Hoppensteadt and Eugene M Izhikevich. Pattern recognition via synchronization in phase-locked loop neural networks. *IEEE Transactions on Neural Networks*, 11(3):734–738, 2000.

[10] Kenneth S. Miller. On the inverse of the sum of matrices. *Mathematics Magazine*, 54(2):pp. 67–72, 1981.
